# Supplementary material for: Generalized Ulam-Hyers-Rassias stability and novel sustainable techniques for dynamical analysis of global warming impact on ecosystem
Source: Sci Rep. 2023 Dec 17;13:22441. doi: 10.1038/s41598-023-49806-7 (PMC10725897; doi:10.1038/s41598-023-49806-7)
Supplement: Supplementary file 1 — Supplementary Information. [file 41598_2023_49806_MOESM1_ESM.zip › Revised Manuscript (1)/Revised Manuscript/Revised Paper.pdf]

Generalized Ulam-Hyers-Rassias stability and novel sustainable techniques for dynamical analysis of global warming impact on ecosystem

Muhammad Farman[a,b,c,*], Aamir Shehzad[c], Kottakkaran Sooppy Nisar[d], Evren Hincal[a], Ali Akgul[e], Ahmed Muhammad Hassan[f]

[a]*Faculty of Arts and Science, Department of Mathematics, Near East University, Cyprus, Turkey*
[b]*Department of Computer Science and Mathematics, Lebanese American University, 1107-2020, Beirut, Lebanon.*
[c]*Institute of Mathematics, Khwaja Fareed University of Engineering and Information Technology, Rahim Yar Khan, Pakistan.*
[d]*Department of Mathematics, College of Science and Humanities in Alkharj, Prince Sattam Bin Abdulaziz University, Alkharj 11942, Saudi Arabia.*
[e]*Faculty of Arts and Science, Department of Mathematics, SIIRT University, Cyprus, Turkey*
[f]*Faculty of engineering, Future University, New Cairo, Egypt*

Abstract

Marine structure changes as a result of climate change, with potential biological implications for human societies and marine ecosystems. These changes include changes in temperatures, flow, discrimination, nutritional inputs, oxygen availability, and acidification of the ocean. In this study, a fractional-order model is constructed using the Caputo fractional operator, which singular and nol-local kernel. A model examines the effects of accelerating global warming on aquatic ecosystems while taking into account variables that change over time, such as the environment and organisms. The positively invariant area also demonstrates positive, bounded solutions of the model treated. The equilibrium states for the occurrence and extinction of fish populations are derived for a feasible solution of the system. We also used fixed-point theorems to analyze the existence and uniqueness of the model. The generalized Ulam-Hyers-Rassias function is used to analyze the stability of the system. To study the impact of the fractional operator through computational simulations, results are generated employing a two-step Lagrange polynomial in the generalized version for the power law kernel and also compared the results with an exponential law and Mittag Leffler kernel. We also produce graphs of the model at various fractional derivative orders to illustrate the important influence that the fractional order has on the different classes of the model with the memory effects of the fractional operator. To help with the oversight of fisheries, this research builds mathematical connections between the natural world and aquatic ecosystems.

Keywords: Climate Change; Marine Ecosystem; Caputo Operator; Modeling; Generalized Ulam-Hyers-Rassias stability.

1. Introduction

Due to its potential negative impacts on society and the environment, global warming continues to be an area of discussion and controversy. Climate change is anticipated to have a significant impact on the ocean, which makes up two-thirds of the land area of the Earth and serves as a thermal capacitor. Given the size of the ocean's ecology, the effects of global warming on it could have severe repercussions that are on par with or even worse compared to the effects of worldwide flooding [1]. In ocean currents and other bodies of water, small organisms called plankton drift freely. They are composed of

*

phytoplankton, which are primary producers that transform energy from dissolved carbon dioxide gas, inorganic compounds, and phytoplankton into carbohydrates. Instead, zooplankton are tiny creatures that consume other types of plankton. Many different creatures, such as mussels, fish, birds, and mammals, consume plankton, which is the initial element in the food web in the marine environment. Both zooplankton and phytoplankton, such as jellyfish and acetes, are harvested for human consumption [2]. Microscopic phytoplankton increases the ocean's capacity to dissolve carbon dioxide and drain carbon from surface waters to deeper layers by converting dissolved carbon dioxide into organic molecules through photosynthesis. This biological mechanism is key to comprehending the prospects of environmental change as well as is an important part of the natural carbon cycle on the planet's surface [3]. Temperature variations throughout the year, water column interaction, supply, and consumption all have an impact on how they behave. These variables can be modified by climate, changing the taxonomic composition, seasonal dynamics, and phytoplankton structure. Climate affects phytoplankton in two ways: directly by physiological processes and indirectly by the supply of nutrients, stratification of the water column, and heterotrophic feeding. These changes affect numerous processes [4]. The majority of carbon dioxide and oxygen are produced and absorbed by marine ecosystems. High levels of greenhouse gases cause the oceans to warm quickly and are contributing to global warming. Due to the disruption of the bicarbonate buffer, which keeps ocean acidity between 7.5 and 8.4, this harm has an impact on planktonic species and fisheries resources. Rapid global warming is raising atmospheric carbon dioxide levels, creating an ocean climate that is unfavorable and resulting in decreased plankton and fish populations in marine ecosystems. If this keeps up, by the end of this century a sizable section of marine ecosystems might be eliminated or degraded [5]. In the past 60 years, the Indian Ocean's marine fish and phytoplankton populations have declined by 20% due to global warming. If the problem remains, fish and plankton populations may drop to 50-90% of their current levels, creating an ecological desert and lowering production [6]. By the end of the twenty-first century, the tropical Pacific might experience a temperature increase of more than 3C, which would threaten between 50 and 80 percent of marine species, especially plankton [7].

The movement of people from rural regions is speeding up urbanization trends, causing population density to rise and altering the quality of life globally. This results in environmental contamination, which harms the land, water, and air while ruining the natural world and changing the climate in urban areas [8]. Global environmental changes are a result of human activity, which started with the Industrial Revolution in the 1750s. Before industrialization, carbon dioxide emissions were minimal, but the end of the Ice Age and the Industrial Revolution resulted in a considerable rise in greenhouse gas concentrations [9]. Carbon dioxide storage and capture are potential ways to stop ocean acidification and global warming. On the other hand, leakage from storage structures can hasten acidification, which might have an impact on environmental bacteria. To comprehend the impacts of carbon dioxide, a review of how various bacteria are affected by carbon dioxide is required [10]. The environment is a battleground for living things, and after the beginning of the industrial era, emissions of greenhouse gases have considerably increased. Since the last 0.8 million years, the average atmospheric carbon dioxide concentration has increased from 280.01 to above 380 ppmv, surpassing that record [11]. The most distant continent, Antarctica, is subject to adverse effects from human activities, such as excessive harvesting, environmental impact, and the invasion of alien species. If these problems aren't addressed at the same time, the marine ecosystems in Antarctica will deteriorate and become more similar to other marine ecosystems in terms of substance, structure, and mechanisms [12]. Warming elevations and severe weather events lead to epidemics, even if vector-borne infectious illnesses may not have the most negative effects on health. These factors, such as frequent rainstorms, rodent infestations, and tainted water, render mosquito-borne parasite and viral infections climate-sensitive [13].

The consequences of global changes in climate on coastal biodiversity and marine ecosystems are extensively studied in the literature [14, 15], with multiple publications [16] statistically detailing

possible effects on marine ecosystems and coastline aquatic organisms. Mathematical modeling and esoteric mathematics are included in the field of mathematics. With the use of mathematical ideas and hypotheses, it is simple to assess the progress of the task, the procedure, the forecasts, and the results. As a result, scientists rely heavily on mathematics today [17]. In several publications, mathematical modeling is used to examine how global warming would affect marine ecosystems. [18] offered a stochastic mathematical model, while [1, 19] proposed a deterministic mathematical model to explain the effects of rising temperatures on aquatic ecosystems. Some papers' writers used statistical-based evaluation to carry out their research [20], while others used literature-based assessment to demonstrate their findings [21].

The capacity of fractional calculus to analyze genetic changes and their effects on the dynamic operations of physical structures has drawn attention. While the fractional order concerns include integration and contour differentiation, these non-local aspects help us grasp practical issues like memory characterization and genetic qualities [22]. Numerous fractional operators are employed in the mathematical modelling of problems that arise in the actual world [23]-[26]. Sekerci and Ozarslan [27] investigated the consequences of predation on the oxygen-plankton system by analyzing a fractional model of oxygen, phytoplankton, and zooplankton dynamics within the Caputo sense. The process of photosynthesis and the generation of oxygen in phytoplankton were studied by researchers [28]. They concentrated on the Caputo fractional derivative and compared the outcomes to the integer-ordered derivative. To understand how nutrients, hazardous phytoplankton, and zooplankton collaborate, a fractional-order mathematical model with a delay in time was developed in [29]. The major goal was to investigate how time latency and fractional order affected the ecology. Another study [30] used a fear function together with a Holling type II function to describe the various prey and predator organisms to explore the influence of memory on interactions between predators and prey in the context of global warming. The Caputo derivative and exponential decay function were used to examine how global warming affects both prey and predators. Bonyah [31] investigated a fractional-order model with two controls for nutrients, phytoplankton, and zooplankton. A broad formulation problem with state and adjoint equations, like a fractional optimal control issue. is provided in left fractional derivative terms. The forward-backward sweep approach, which is employed to address the fractional optimum control issue, was created using the Adams-type predictor-corrector technique. Kumar et al.[32] looked into an ecological model that includes the production of oxygen during photosynthesis, plankton apnea, and the effects of zooplankton predation on phytoplankton. This model is a fractional derivative and considers all three components of the food chain. Using a generalized Liouville-Caputo type fractional derivative, they have created a system of three non-integer order differential equations. They started by introducing the issue more conceptually and then employed a current fractional numerical method to demonstrate it experimentally. A fractional-order nonlinear mathematical model was put up in [33] to examine the behavior of climate change using the Caputo operator. The model explained the effects of declining water quality brought on by greenhouse gases on populations of aquatic animals. The model looked at equilibrium locations and talked about how equilibria can be asymptotically stable. The model's singular solution was established, and the numerical solution was discovered using a generalized predictor-corrector technique. As a result of the above debate, we analyze a fractional-order mathematical representation with time-dependent parameters to show the fast global warming's prospective impacts on marine ecosystems. The Caputo fractional derivative notion performs better than ordinary integer-order derivatives. This distinguishes our concept and process from the earlier model that has been provided up to this point.

A generalized version of the model and a summary of the description of the proposed model are provided in Section "2". Furthermore, the theoretical background of the suggested fractional operator is explored. Section 3" deals with the qualitative analysis of the proposed system. The numerical solutions to the suggested fractional-order model with power law kernel are provided in Section "4". In Sections "5" and "6," the numerical simulations, results, and conclusions are addressed.

2. Environment Management Model with Caputo fractional derivative

We take into consideration the ecological maintenance model with time-varying characteristics described in [5]. A model that explores how global warming would affect aquatic ecosystems as a result of the fast emitted greenhouse gases (GGs) caused by humankind. The four main components of the diverse system are the density of environmental GGs, $\mathbf{G}(t)$, which are quickly released through different sources; rising temperature in the atmosphere, $\mathbf{T}(t)$, which grows correspondingly with rising levels of environmental GGs along with is the cause of global warming; the density of planktonic population in marine ecosystems, $\mathbf{P}(t)$, which is continually at risk by rising temperature and GGs concentration; and the fish population density in marine environments, $\mathbf{F}(t)$, which is likewise declining in quantity because of increasing global warming, acidification, a lack of saturation oxygen, and a lack of planktonic species.

2.1. Model's Assumptions

- The usual growth rates of $\mathbf{G}$ and $\mathbf{T}$ are g_1 and g_2, respectively. However, in not having any of the negative effects of GGs and global warming, $\mathbf{P}$ and $\mathbf{F}$ expand at their usual rates of g_3 and g_4, respectively.

- In marine ecosystems, fish emit saturated Carbon-dioxide, which marginally raises the concentration of GGs. The rise in GGs concentration caused by the fish population is shown in this case by $\varphi_1\mathbf{GF}$.

- The photosynthesis carried out by phytoplankton in marine ecosystems lowers the levels of GGs in the atmosphere. Planktonic population GGs absorption is represented by $\varphi_2\mathbf{GP}$.

- The density of GGs, defined by $\varphi_3\mathbf{T}$, rises as a result of natural disasters brought on by climate change, such as droughts and forest fires.

- In accordance with the density of ambient GGs, the temperature of the atmosphere rises. A rise in the temperature of the atmosphere brought on by rising GGs is shown here in the form of $\lambda_1\mathbf{GT}$.

- The photosynthesis of aquatic plankton is influenced by temperature, and this enables them to counteract increasing temperatures. Planktonic population absorption of ambient temperature is represented by the symbol $\lambda_2\mathbf{PT}$.

- All living beings have an ultimate carrying capacity, therefore supposing a constant level, $\alpha(0 < \alpha < 1)$, dissolved Carbon-dioxide saturation is essential to their lifespan.

- We choose Q_1 and Q_2 as the carrying capacities for the fish population and the planktonic population, respectively. $\frac{g_3}{Q_1}$ and $\frac{g_4}{Q_2}$ are the appropriate decomposition rates.

- While high concentrations impede development through increased plankton respiration and decreased oxygen dispersion density, they do enhance the overall density of marine plankton. This inhibits the growth of marine fisheries and causes a dissolved oxygen shortfall when paired with saturated Carbon-dioxide. As a result, a rise in planktonic population resulting from Carbon-dioxide absorption is represented by "$\frac{\omega_1\mathbf{P}}{\alpha+\mathbf{G}}$", while a fish population reduction caused by an excessive amount of dissolved Carbon-dioxide is shown by $\frac{\xi_2\mathbf{F}}{\alpha+\mathbf{G}}$.

- Plankton and fish density are continuously declining as a result of acidification and rising temperatures harming marine ecosystems. Planktonic population reduction caused by warming is shown by $\omega_2\mathbf{PT}$, planktonic population reduction linked to acidity is shown by $\omega_4\mathbf{GP}$, and the fish density demise in response to rising temperatures is shown by $\xi_3\mathbf{TF}$.

- In marine ecosystems, fish populations serve as predators and planktonic populations as prey. Therefore, we infer that the decline in the planktonic population due to fish population predation is represented by $\omega_3\mathbf{FP}$. And $\xi_1\mathbf{FP}$ represents the growth in fish density caused by their consumption of planktonic organisms.

Here, we use the nonlinear fractional-order system to define the aforementioned description.

$$\begin{aligned}
{}_0^C D_t^\beta \mathbf{G}(t) &= g_1\mathbf{G} + \gamma_1\mathbf{GF} - \gamma_2\mathbf{GP} + \gamma_3\mathbf{T},\\
{}_0^C D_t^\beta \mathbf{T}(t) &= g_2\mathbf{T} + \lambda_1\mathbf{GT} - \lambda_2\mathbf{PT},\\
{}_0^C D_t^\beta \mathbf{P}(t) &= g_3\mathbf{P}\big(1 - \frac{\mathbf{P}}{Q_1}\big) + \frac{\omega_1\mathbf{P}}{\alpha + \mathbf{G}} - \omega_2\mathbf{PT} - \omega_3\mathbf{FP} - \omega_4\mathbf{GP},\\
{}_0^C D_t^\beta \mathbf{F}(t) &= g_4\mathbf{F}\big(1 - \frac{\mathbf{F}}{Q_2}\big) + \xi_1\mathbf{FP} - \frac{\xi_2\mathbf{F}}{\alpha + \mathbf{G}} - \xi_3\mathbf{TF}.
\end{aligned} \tag{1}$$

Where ${}^C D^\beta$ represents the Caputo derivative of order $0 < \beta \le 1$. The corresponding nonnegative initial conditions are such that

$$\mathbf{G}(0),\ \mathbf{T}(0),\ \mathbf{P}(0),\ \mathbf{F}(0) \ge 0. \tag{2}$$

Now we'll go through some recent and relevant calculus results.

Definition 2.1. *[34] The Caputo derivative of a differentiable function $\zeta(t)$ to order $\beta \in (0,1)$ with beginning point, $t = 0$, is given by*

$${}_0^C D_t^\beta \zeta(t) = \frac{1}{\Gamma(1-\beta)} \int_0^t \frac{\zeta'(\nu)}{(t-\nu)^\beta} d\nu. \tag{3}$$

Definition 2.2. *If $\zeta(t)$ is an integrable function with $0 < \beta < 1$, the fractional integral is specified as follows [35]:*

$${}_0^C I_t^\beta \zeta(t) = \frac{1}{\Gamma(\beta)} \int_0^t \frac{\zeta(\nu)}{(t-\nu)^{1-\beta}} d\nu. \tag{4}$$

Remark 2.1. *A fixed point $\tau^\star$ is regarded as the equilibrium point of the Caputo system*

$${}_0^C D_t^\beta \zeta(t) = \zeta(t, \tau(t)), \qquad \beta \in (0,1) \tag{5}$$

if and only if $\zeta(t, \tau^\star) = 0$.

Lemma 2.1. *[36] Consider that the function $\zeta(t) \in \mathbb{R}^+$ is differentiable. Then for $\beta \in (0,1)$,*

$${}_0^C D_t^\beta \Big(\zeta(t) - \zeta^* - \zeta^* \ln \frac{\zeta(t)}{\zeta^*}\Big) \le \Big[1 - \frac{\zeta^*}{\zeta(t)}\Big] {}_0^C D_t^\beta \zeta(t), \qquad \forall t \ge 0. \tag{6}$$

Lemma 2.2. *[38, 39] Consider $\beta \in \mathbb{R}^+$, $\eta_1(t)$, and $\eta_2(t)$ demonstrate positive functions and $\eta_3(t)$ denote an increasing as well as positive function for $t \in [0, \mathbb{T}]$, $\mathbb{T} > 0$, and $\eta_3(t) \le m$, while m is a constant value. Suppose*

$$\eta_1 \le \eta_2 + \eta_3(t) \int_0^{\mathbb{T}} (t-\nu)^{\beta-1} \eta_1(\nu) d\nu, \tag{7}$$

then

$$\eta_1 \le \eta_2 \mathrm{E}_\beta \Big[\eta_3(t) \frac{\pi \mathbb{T}^\beta}{\Gamma(1-\beta)\sin(\beta\pi)}\Big]. \tag{8}$$

3. Qualitative Analysis of the Model

3.1. Well-posedness and Positively Invariant Region

Here, we examine the conditions necessary for a system to produce favorable results while taking into account realistic real-world scenarios.

Theorem 3.1. *For every $t \geq 0$, the system (1)'s solutions are not negative.*

Proof. Here, we define a norm

$$\|\mathrm{H}\|_\infty = \sup_{t\in D_{\mathrm{H}}} |\mathrm{H}(t)|, \tag{9}$$

where D_{H} is the domain of H.

$$\begin{aligned} {}_0^C D_t^\beta \mathbf{G}(t) &= g_1\mathbf{G} + \gamma_1\mathbf{GF} - \gamma_2\mathbf{GP} + \gamma_3\mathbf{T} \;\geq\; -\{\gamma_2\mathbf{P} - \gamma_1\mathbf{F}\}\mathbf{G} \\ &\geq\; -\{\gamma_2 \sup_{t\in D_{\mathbf{P}}}|\mathbf{P}| - \gamma_1 \sup_{t\in D_{\mathbf{F}}}|\mathbf{F}|\}\mathbf{G} \;=\; -\{\gamma_2\|\mathbf{P}\|_\infty - \gamma_1\|\mathbf{F}\|_\infty\}\mathbf{G} \\ \Rightarrow\; \mathbf{G}(t) &= \mathbf{G}(0)\, e^{-\{\gamma_2\|\mathbf{P}\|_\infty \,-\, \gamma_1\|\mathbf{F}\|_\infty\}t}, \quad \textit{for all } t \geq 0. \end{aligned} \tag{10}$$

$$\begin{aligned} {}_0^C D_t^\beta \mathbf{T}(t) &= g_2\mathbf{T} + \lambda_1\mathbf{GT} - \lambda_2\mathbf{PT} \;\geq\; -\{\lambda_2\mathbf{P} - \lambda_1\mathbf{G}\}\mathbf{T} \\ &\geq\; -\{\lambda_2 \sup_{t\in D_{\mathbf{P}}}|\mathbf{P}| - \lambda_1 \sup_{t\in D_{\mathbf{G}}}|\mathbf{G}|\}\mathbf{T} \;=\; -\{\lambda_2\|\mathbf{P}\|_\infty - \lambda_1\|\mathbf{G}\|_\infty\}\mathbf{T} \\ \Rightarrow\; \mathbf{T}(t) &= \mathbf{T}(0)\, e^{-\{\lambda_2\|\mathbf{P}\|_\infty \,-\, \lambda_1\|\mathbf{G}\|_\infty\}t}, \quad \textit{for all } t \geq 0. \end{aligned} \tag{11}$$

$$\begin{aligned} {}_0^C D_t^\beta \mathbf{P}(t) &= g_3\mathbf{P}\big(1 - \frac{\mathbf{P}}{Q_1}\big) + \frac{\omega_1\mathbf{P}}{\alpha + \mathbf{G}} - \omega_2\mathbf{PT} - \omega_3\mathbf{FP} - \omega_4\mathbf{GP} \;\geq\; -\Big\{\omega_2\mathbf{T} + \omega_3\mathbf{F} + \omega_4\mathbf{G}\Big\}\mathbf{P} \\ &\geq\; -\Big\{\omega_2 \sup_{t\in D_{\mathbf{T}}}|\mathbf{T}| + \omega_3 \sup_{t\in D_{\mathbf{F}}}|\mathbf{F}| + \omega_4 \sup_{t\in D_{\mathbf{G}}}|\mathbf{G}|\Big\}\mathbf{P} \;=\; -\Big\{\omega_2\|\mathbf{T}\|_\infty + \omega_3\|\mathbf{F}\|_\infty + \omega_4\|\mathbf{G}\|_\infty\Big\}\mathbf{P} \\ \Rightarrow\; \mathbf{P}(t) &= \mathbf{P}(0)\, e^{-\big\{\omega_2\|\mathbf{T}\|_\infty + \omega_3\|\mathbf{F}\|_\infty + \omega_4\|\mathbf{G}\|_\infty\big\}t}, \quad \textit{for all } t \geq 0. \end{aligned} \tag{12}$$

$$\begin{aligned} {}_0^C D_t^\beta \mathbf{F}(t) &= g_4\mathbf{F}\big(1 - \frac{\mathbf{F}}{Q_2}\big) + \xi_1\mathbf{FP} - \frac{\xi_2\mathbf{F}}{\alpha + \mathbf{G}} - \xi_3\mathbf{TF} \;\geq\; -\Big\{\xi_3\mathbf{T} - \xi_1\mathbf{P}\Big\}\mathbf{F} \\ &\geq\; -\Big\{\xi_3 \sup_{t\in D_{\mathbf{T}}}|\mathbf{T}| - \xi_1 \sup_{t\in D_{\mathbf{P}}}|\mathbf{P}|\Big\}\mathbf{F} \;=\; -\Big\{\xi_3\|\mathbf{T}\|_\infty - \xi_1\|\mathbf{P}\|_\infty\Big\}\mathbf{F} \\ \Rightarrow\; \mathbf{F}(t) &= \mathbf{F}(0)\, e^{-\big\{\xi_3\|\mathbf{T}\|_\infty - \xi_1\|\mathbf{P}\|_\infty\big\}t}, \quad \textit{for all } t \geq 0. \end{aligned} \tag{13}$$

While the positive solutions under fractional Caputo derivative are [37]:

$$\begin{aligned} \mathbf{G}(t) &\geq \mathbf{G}(0)\, E_\beta\Big(-\{\gamma_2\|\mathbf{P}\|_\infty \,-\, \gamma_1\|\mathbf{F}\|_\infty\}t^\beta\Big), \\ \mathbf{T}(t) &\geq \mathbf{T}(0)\, E_\beta\Big(-\{\lambda_2\|\mathbf{P}\|_\infty \,-\, \lambda_1\|\mathbf{G}\|_\infty\}t^\beta\Big), \\ \mathbf{P}(t) &\geq \mathbf{P}(0)\, E_\beta\Big(-\{\omega_2\|\mathbf{T}\|_\infty + \omega_3\|\mathbf{F}\|_\infty + \omega_4\|\mathbf{G}\|_\infty\}t^\beta\Big), \\ \mathbf{F}(t) &\geq \mathbf{F}(0)\, E_\beta\Big(-\{\xi_3\|\mathbf{T}\|_\infty - \xi_1\|\mathbf{P}\|_\infty\}t^\beta\Big), \end{aligned} \tag{14}$$

for all $t \geq 0$. Where E_β represents Mittag-Leffler function. □

Theorem 3.2. *The recommended solution of the environment management model (1) is distinct and limited in R_+^4 given straight-line constraints.*

Proof: We have got

$$
\begin{aligned}
{}_0^C D_t^{\beta}\mathbf{G}(t)\big|_{\mathbf{G}=0} &= \gamma_3\mathbf{T} \geq 0,\\
{}_0^C D_t^{\beta}\mathbf{T}(t)\big|_{\mathbf{T}=0} &= 0,\\
{}_0^C D_t^{\beta}\mathbf{P}(t)\big|_{\mathbf{P}=0} &= 0,\\
{}_0^C D_t^{\beta}\mathbf{F}(t)\big|_{\mathbf{F}=0} &= 0.
\end{aligned} \tag{15}
$$

The choice of solution can't escape from the hyperplane if $(\mathbf{G}(0), \mathbf{T}(0), \mathbf{P}(0), \mathbf{F}(0)) \in R_{+}^{4}$. The vector field on each hyperplane surrounding the non-negative orthant directs into the domain R_{+}^{4}, making it a positively invariant set.

3.2. Equilibrium Points Analysis

If the system (1)'s left side is set to zero, the two different sorts of equilibrium points are obtained [5] are given here

- The equilibrium point when there are no more fish is $\mathbf{E}^{\circ} = \{\mathbf{G}^{\circ}, \mathbf{T}^{\circ}, \mathbf{P}^{\circ}, \mathbf{F}^{\circ}\}$. Where

$$
\begin{aligned}
\mathbf{G}^{\circ} &= \frac{\gamma_3\mathbf{T}^{\circ}}{\gamma_2\mathbf{P}^{\circ} - g_1},\\
\mathbf{T}^{\circ} &= \frac{\omega_1\lambda_2(g_1 - g_2) + \alpha g_1 g_2 g_3 \gamma_2}{\alpha g_1 \omega_4 \gamma_3 \lambda_2},\\
\mathbf{P}^{\circ} &= \frac{g_2}{\lambda_2} + \frac{g_1(\gamma_3\lambda_2\mathbf{T}^{\circ} + 2\alpha\gamma_2 g_2)}{\lambda_2\gamma_2(\omega_1 - 2\alpha g_1 - \alpha g_1 g_3)},\\
\mathbf{F}^{\circ} &= 0.
\end{aligned} \tag{16}
$$

- The simultaneously completed equilibrium point is $\mathbf{E}^{\bullet} = \{\mathbf{G}^{\bullet},\ \mathbf{T}^{\bullet},\ \mathbf{P}^{\bullet},\ \mathbf{F}^{\bullet}\}$. Where

$$
\begin{aligned}
\mathbf{G}^{\bullet} &= \frac{\gamma_3\mathbf{T}^{\bullet}}{\gamma_2\mathbf{P}^{\bullet} - g_1 - \gamma_1\mathbf{F}^{\bullet}},\\
\mathbf{T}^{\bullet} &= \frac{g_1(g_2 - \lambda_1\mathbf{P}^{\bullet})}{\lambda_1\gamma_3},\\
\mathbf{P}^{\bullet} &= \frac{1}{2\lambda_1}\Big[\frac{\gamma_3 Q_1\omega_3\lambda_2\mathbf{F}^{\bullet}}{\omega_2 g_1 Q_1 - g_3\gamma_3} - \lambda_1(\alpha - 1) - g_2\Big],\\
\mathbf{F}^{\bullet} &= \frac{\alpha\xi_1 {Q_1}^2(\lambda_1 g_3 + g_2\omega_4)}{4 g_3 g_4}.
\end{aligned} \tag{17}
$$

3.3. Solutions' Existence and Uniqueness

Implementing the Banach fixed point theory and Schaefer's fixed point theorem, this section establishes the existence and distinctiveness of a solution to the system (1). Organize the following function:

$$
\begin{aligned}
\mathrm{U}(t, \mathbf{G}, \mathbf{T}, \mathbf{P}, \mathbf{F}) &= g_1\mathbf{G} + \gamma_1\mathbf{GF} - \gamma_2\mathbf{GP} + \gamma_3\mathbf{T},\\
\mathrm{V}(t, \mathbf{G}, \mathbf{T}, \mathbf{P}, \mathbf{F}) &= g_2\mathbf{T} + \lambda_1\mathbf{GT} - \lambda_2\mathbf{PT},\\
\mathrm{W}(t, \mathbf{G}, \mathbf{T}, \mathbf{P}, \mathbf{F}) &= g_3\mathbf{P}\big(1 - \frac{\mathbf{P}}{Q_1}\big) + \frac{\omega_1\mathbf{P}}{\alpha + \mathbf{G}} - \omega_2\mathbf{PT} - \omega_3\mathbf{FP} - \omega_4\mathbf{GP},\\
\mathrm{X}(t, \mathbf{G}, \mathbf{T}, \mathbf{P}, \mathbf{F}) &= g_4\mathbf{F}\big(1 - \frac{\mathbf{F}}{Q_2}\big) + \xi_1\mathbf{FP} - \frac{\xi_2\mathbf{F}}{\alpha + \mathbf{G}} - \xi_3\mathbf{TF}.
\end{aligned} \tag{18}
$$

The Caputo fractional derivative model (1) of order $\beta > 0$ will therefore be subjected to the fractional integral according to its beginning conditions. The second kind of Volterra-integral equations that are produced as a consequence of the procedure provide the answer to the proposed model (1).

$$\begin{aligned}
\mathbf{G}(t)-\mathbf{G}(0) &= \frac{1}{\Gamma(\beta)}\int_0^t (t-\nu)^{\beta-1}\mathrm{U}(\nu,\mathbf{G}(\nu))d\nu,\\
\mathbf{T}(t)-\mathbf{T}(0) &= \frac{1}{\Gamma(\beta)}\int_0^t (t-\nu)^{\beta-1}\mathrm{V}(\nu,\mathbf{T}(\nu))d\nu,\\
\mathbf{P}(t)-\mathbf{P}(0) &= \frac{1}{\Gamma(\beta)}\int_0^t (t-\nu)^{\beta-1}\mathrm{W}(\nu,\mathbf{P}(\nu))d\nu,\\
\mathbf{F}(t)-\mathbf{F}(0) &= \frac{1}{\Gamma(\beta)}\int_0^t (t-\nu)^{\beta-1}\mathrm{X}(\nu,\mathbf{F}(\nu))d\nu.
\end{aligned} \tag{19}$$

In order for $(\mathfrak{D}, \|.\|)$ to be the Banach space and $\mathrm{K}^1([0,\mathbb{T}])$ to be the Banach space comprising all continuous functions established in $[0,\mathbb{T}] \to \mathfrak{D}$ formed with Chebyshev norm, the functions $(\mathbf{G},\mathbf{T},\mathbf{P},\mathbf{F}) : [0,\mathbb{T}]\times\mathfrak{D}\to\mathfrak{D}$ is considered to be continuous. The Lipschitz condition is met by the continuous functions $\mathbf{G}$, $\mathbf{T}$, $\mathbf{P}$, and $\mathbf{F}$ if

$$\sup_{t\in(0,\mathbb{T}]}\|\mathbf{G}\|\le\Psi_1, \qquad \sup_{t\in(0,\mathbb{T}]}\|\mathbf{T}\|\le\Psi_2, \qquad \sup_{t\in(0,\mathbb{T}]}\|\mathbf{P}\|\le\Psi_3, \qquad \sup_{t\in(0,\mathbb{T}]}\|\mathbf{F}\|\le\Psi_4. \tag{20}$$

Therefore

$$\begin{aligned}
\|\mathrm{U}(\mathbf{G}_1)-\mathrm{U}(\mathbf{G}_2)\| &= \big\|\big[(g_1+\gamma_1\mathbf{F}-\gamma_2\mathbf{P})\mathbf{G}_1+\gamma_3\mathbf{T}\big]-\big[(g_1+\gamma_1\mathbf{F}-\gamma_2\mathbf{P})\mathbf{G}_2+\gamma_3\mathbf{T}\big]\big\|\\
&= \big\|g_1(\mathbf{G}_1-\mathbf{G}_2)+\gamma_1\mathbf{F}(\mathbf{G}_1-\mathbf{G}_2)-\gamma_2\mathbf{P}(\mathbf{G}_1-\mathbf{G}_2)\big\|\\
&\le g_1\|\mathbf{G}_1-\mathbf{G}_2\|+\gamma_1\sup_{t\in(0,\mathbb{T}]}\|\mathbf{F}\|\|\mathbf{G}_1-\mathbf{G}_2\|+\gamma_2\sup_{t\in(0,\mathbb{T}]}\|\mathbf{P}\|\|\mathbf{G}_1-\mathbf{G}_2\|\\
&\le \varpi_{\mathrm{U}}\|\mathbf{G}_1-\mathbf{G}_2\|,
\end{aligned} \tag{21}$$

where $\varpi_{\mathrm{U}} = (g_1+\gamma_1\Psi_4-\gamma_2\Psi_3) > 0$.

$$\begin{aligned}
\|\mathrm{V}(\mathbf{T}_1)-\mathrm{V}(\mathbf{T}_2)\| &= \big\|(g_2+\lambda_1\mathbf{G}-\lambda_2\mathbf{P})\mathbf{T}_1-(g_2+\lambda_1\mathbf{G}-\lambda_2\mathbf{P})\mathbf{T}_2\big\|\\
&= \big\|g_2(\mathbf{T}_1-\mathbf{T}_2)+\lambda_1\mathbf{G}(\mathbf{T}_1-\mathbf{T}_2)-\lambda_2\mathbf{P}(\mathbf{T}_1-\mathbf{T}_2)\big\|\\
&\le g_2\|\mathbf{T}_1-\mathbf{T}_2\|+\lambda_1\sup_{t\in(0,\mathbb{T}]}\|\mathbf{G}\|\|\mathbf{T}_1-\mathbf{T}_2\|+\lambda_2\sup_{t\in(0,\mathbb{T}]}\|\mathbf{P}\|\|\mathbf{T}_1-\mathbf{T}_2\|\\
&\le \varpi_{\mathrm{V}}\|\mathbf{T}_1-\mathbf{T}_2\|,
\end{aligned} \tag{22}$$

where $\varpi_{\mathrm{V}} = (g_1+\lambda_1\Psi_1-\lambda_2\Psi_3) > 0$.

$$\begin{aligned}
&\|\mathrm{W}(\mathbf{P}_1)-\mathrm{W}(\mathbf{P}_2)\|\\
&= \Big\|\Big[g_3\big(1-\frac{\mathbf{P}}{Q_1}\big)+\frac{\omega_1}{\alpha+\mathbf{G}}-\omega_2\mathbf{T}-\omega_3\mathbf{F}-\omega_4\mathbf{G}\Big]\mathbf{P}_1-\Big[g_3\big(1-\frac{\mathbf{P}}{Q_1}\big)+\frac{\omega_1}{\alpha+\mathbf{G}}-\omega_2\mathbf{T}-\omega_3\mathbf{F}-\omega_4\mathbf{G}\Big]\mathbf{P}_2\Big\|\\
&= \Big\|g_3\big(1-\frac{\mathbf{P}}{Q_1}\big)(\mathbf{P}_1-\mathbf{P}_2)+\frac{\omega_1}{\alpha+\mathbf{G}}(\mathbf{P}_1-\mathbf{P}_2)-\omega_2\mathbf{T}(\mathbf{P}_1-\mathbf{P}_2)-\omega_3\mathbf{F}(\mathbf{P}_1-\mathbf{P}_2)-\omega_4\mathbf{G}(\mathbf{P}_1-\mathbf{P}_2)\Big\|\\
&\le g_3\|(\mathbf{P}_1-\mathbf{P}_2)\|+\frac{g_3}{Q_1}\sup_{t\in(0,\mathbb{T}]}\|\mathbf{P}\|\,\|(\mathbf{P}_1-\mathbf{P}_2)\|+\frac{\omega_1}{\alpha+\sup_{t\in(0,\mathbb{T}]}\|\mathbf{G}\|}\|\mathbf{P}_1-\mathbf{P}_2\|\\
&\qquad+\omega_2\sup_{t\in(0,\mathbb{T}]}\|\mathbf{T}\|\,\|\mathbf{P}_1-\mathbf{P}_2\|+\omega_3\sup_{t\in(0,\mathbb{T}]}\|\mathbf{F}\|\,\|\mathbf{P}_1-\mathbf{P}_2\|+\omega_4\sup_{t\in(0,\mathbb{T}]}\|\mathbf{G}\|\,\|\mathbf{P}_1-\mathbf{P}_2\|\\
&\le \varpi_{\mathrm{W}}\|\mathbf{P}_1-\mathbf{P}_2\|,
\end{aligned} \tag{23}$$

where $\varpi_{\mathrm{W}} = \left\{ g_3 + \frac{g_3}{Q_1}\Psi_3 + \frac{\omega_1}{\alpha+\Psi_1} + \omega_2\Psi_2 + \omega_3\Psi_4 + \omega_4\Psi_1 \right\} > 0$.

$$
\begin{aligned}
\|\mathrm{X}(\mathbf{F}_1) - \mathrm{X}(\mathbf{F}_2)\| &= \left\| \left[g_4\big(1 - \frac{\mathbf{F}}{Q_2}\big) + \xi_1\mathbf{P} - \frac{\xi_2}{\alpha+\mathbf{G}} - \xi_3\mathbf{T} \right]\mathbf{F}_1 - \left[g_4\big(1 - \frac{\mathbf{F}}{Q_2}\big) + \xi_1\mathbf{P} - \frac{\xi_2}{\alpha+\mathbf{G}} - \xi_3\mathbf{T} \right]\mathbf{F}_2 \right\| \\
&= \left\| g_4\big(1 - \frac{\mathbf{F}}{Q_2}\big)(\mathbf{F}_1 - \mathbf{F}_2) + \xi_1\mathbf{P}(\mathbf{F}_1 - \mathbf{F}_2) - \frac{\xi_2}{\alpha+\mathbf{G}}(\mathbf{F}_1 - \mathbf{F}_2) - \xi_3\mathbf{T}(\mathbf{F}_1 - \mathbf{F}_2) \right\| \\
&\leq g_4\|\mathbf{F}_1 - \mathbf{F}_2\| + \frac{g_4}{Q_2}\sup_{t\in(0,\mathbb{T}]}\|\mathbf{F}\|\,\|\mathbf{F}_1 - \mathbf{F}_2\| + \xi_1\sup_{t\in(0,\mathbb{T}]}\|\mathbf{P}\|\|\mathbf{F}_1 - \mathbf{F}_2\| \\
&\qquad + \frac{\xi_2}{\alpha + \sup_{t\in(0,\mathbb{T}]}\|\mathbf{G}\|}\|\mathbf{F}_1 - \mathbf{F}_2\| + \xi_3\sup_{t\in(0,\mathbb{T}]}\|\mathbf{T}\|\|\mathbf{F}_1 - \mathbf{F}_2\| \\
&\leq \varpi_{\mathrm{X}}\|\mathbf{F}_1 - \mathbf{F}_2\|,
\end{aligned} \tag{24}
$$

where $\varpi_{\mathrm{X}} = \left\{ g_4 + \frac{g_4}{Q_2}\Psi_4 + \xi_1\Psi_3 + \frac{\xi_2}{\alpha+\Psi_1} + \xi_3\Psi_2 \right\} > 0$.

Theorem 3.3. *Suppose that the functions $(\mathbf{G},\mathbf{T},\mathbf{P},\mathbf{F}) : [0,\mathbb{T}] \times \mathfrak{D} \to \mathfrak{D}$ are continuous and ensure the Lipschitz condition. The system (1) has a unique solution if*

$$
(\mathbf{G},\mathbf{T},\mathbf{P},\mathbf{F})\frac{\Gamma(1-\beta)\sin(\beta\pi)\mathbb{T}^\beta}{\beta\pi} < 1. \tag{25}
$$

Proof. Construct the mapping $\mathrm{J} : \mathrm{K}^1([0,\mathbb{T}],\mathfrak{D}) \to \mathrm{K}^1([0,\mathbb{T}],\mathfrak{D})$, where $\mathrm{J} \in (\mathbf{G},\mathbf{T},\mathbf{P},\mathbf{F}) : [0,\mathbb{T}] \times \mathfrak{D} \to \mathfrak{D}$. Following (21)-(24) and for all $\{(\mathbf{G}_1,\mathbf{G}_2),(\mathbf{T}_1,\mathbf{T}_2),(\mathbf{P}_1,\mathbf{P}_2),(\mathbf{F}_1,\mathbf{F}_2)\} \in \mathrm{K}^1([0,\mathbb{T}],\mathfrak{D})$ and $t \in [0,\mathbb{T}]$, we have

$$
\begin{aligned}
&\|\mathrm{J}(\mathbf{G}_1(t)) - \mathrm{J}(\mathbf{G}_2(t))\| \\
&\quad = \left\| \left[\mathbf{G}(0) + \frac{1}{\Gamma(\beta)}\int_0^{\mathbb{T}} (t-v)^{\beta-1}\mathrm{U}(v,\mathbf{G}_1(v))dv\right] - \left[\mathbf{G}(0) + \frac{1}{\Gamma(\beta)}\int_0^{\mathbb{T}} (t-v)^{\beta-1}\mathrm{U}(v,\mathbf{G}_2(v))dv\right] \right\| \\
&\quad \leq \frac{1}{\Gamma(\beta)}\int_0^{\mathbb{T}} (t-v)^{\beta-1}\|\mathrm{U}(v,\mathbf{G}_1(v)) - \mathrm{U}(v,\mathbf{G}_2(v))\|dv \\
&\quad \leq \frac{\varpi_{\mathrm{U}}}{\Gamma(\beta)}\int_0^{\mathbb{T}} (t-v)^{\beta-1}\|\mathbf{G}_1(v) - \mathbf{G}_2(v)\|dv \;\leq\; \frac{\varpi_{\mathrm{U}}\mathbb{T}^\beta}{\Gamma(\beta+1)}\|\mathbf{G}_1 - \mathbf{G}_2\|_{\mathrm{K}^1},
\end{aligned} \tag{26}
$$

Similarly, we find

$$
\|\mathrm{J}(\mathbf{T}_1(t)) - \mathrm{J}(\mathbf{T}_2(t))\| \;\leq\; \frac{\varpi_{\mathrm{V}}\mathbb{T}^\beta}{\Gamma(\beta+1)}\|\mathbf{T}_1 - \mathbf{T}_2\|_{\mathrm{K}^1}. \tag{27}
$$

$$
\|\mathrm{J}(\mathbf{P}_1(t)) - \mathrm{J}(\mathbf{P}_2(t))\| \;\leq\; \frac{\varpi_{\mathrm{W}}\mathbb{T}^\beta}{\Gamma(\beta+1)}\|\mathbf{P}_1 - \mathbf{P}_2\|_{\mathrm{K}^1}. \tag{28}
$$

$$
\|\mathrm{J}(\mathbf{F}_1(t)) - \mathrm{J}(\mathbf{F}_2(t))\| \;\leq\; \frac{\varpi_{\mathrm{X}}\mathbb{T}^\beta}{\Gamma(\beta+1)}\|\mathbf{F}_1 - \mathbf{F}_2\|_{\mathrm{K}^1}. \tag{29}
$$

The fact that the condition $(\mathbf{G},\mathbf{T},\mathbf{P},\mathbf{F})\frac{\Gamma(1-\beta)\sin(\beta\pi)\mathbb{T}^\beta}{\beta\pi} < 1$ is clear from the data. Considering that the parameter J contains a fixed point in $t \in [0,\mathbb{T}]$ as it is a contraction mapping, the Banach contraction mapping concept is applied to demonstrate this. □

Now, we look at the existence of solutions for the system (1) using Schaefer's fixed point theorem.

Theorem 3.4. *Provided that the variables* $(\mathbf{G},\mathbf{T},\mathbf{P},\mathbf{F}) : [0,\mathbb{T}]\times\mathfrak{D}\to\mathfrak{D}$ *are continuous and the fact that the constants* $(\varpi_{U_1},\varpi_{V_1},\varpi_{W_1},\varpi_{X_1}) > 0$ *exist such that*

$$\begin{aligned}
\|U(t,\mathbf{G})\| &\le \varpi_{U_1}(\rho+\|\mathbf{G}\|),\\
\|V(t,\mathbf{T})\| &\le \varpi_{V_1}(\rho+\|\mathbf{T}\|),\\
\|W(t,\mathbf{P})\| &\le \varpi_{W_1}(\rho+\|\mathbf{P}\|),\\
\|X(t,\mathbf{F})\| &\le \varpi_{X_1}(\rho+\|\mathbf{F}\|),
\end{aligned} \tag{30}$$

where ρ *is a random number between 0 and 1, then system (1) has at least one solution.*

Proof. We can infer that the operator J is continuous from above Theorem (3.3). Suppose that $\{\mathbf{G}^{r+1}\}_\infty$, $\{\mathbf{T}^{r+1}\}_\infty$, $\{\mathbf{P}^{r+1}\}_\infty$, and $\{\mathbf{F}^{r+1}\}_\infty$ be sequences such that $\mathbf{G}^{r+1}\to\mathbf{G}^r$, $\mathbf{T}^{r+1}\to\mathbf{T}^r$, $\mathbf{P}^{r+1}\to\mathbf{P}^r$, and $\mathbf{F}^{r+1}\to\mathbf{F}^r$, in $K^1([0,\mathbb{T}],\mathfrak{D})$. For $t\in[0,\mathbb{T}]$, we have

$$\begin{aligned}
\left\|J(\mathbf{G}^{r+1}(t))-J(\mathbf{G}^r(t))\right\| &= \frac{1}{\Gamma(\beta)}\left\|\int_0^t (t-\nu)^{\beta-1}U(\nu,\mathbf{G}^{r+1}(\nu))d\nu-\int_0^t (t-\nu)^{\beta-1}U(\nu,\mathbf{G}^{r}(\nu))d\nu\right\|\\
&\le \frac{1}{\Gamma(\beta)}\int_0^t (t-\nu)^{\beta-1}\left\|U(\nu,\mathbf{G}^{r+1}(\nu))-U(\nu,\mathbf{G}^{r}(\nu))\right\|d\nu\\
&\le \frac{\varpi_{U_1}\mathbb{T}^\beta}{\Gamma(\beta+1)}\left\|\mathbf{G}^{r+1}-\mathbf{G}^r\right\|_{K^1}.
\end{aligned} \tag{31}$$

$$\left\|J(\mathbf{T}^{r+1}(t))-J(\mathbf{T}^r(t))\right\| \le \frac{\varpi_{V_1}\mathbb{T}^\beta}{\Gamma(\beta+1)}\left\|\mathbf{T}^{r+1}-\mathbf{T}^r\right\|_{K^1}. \tag{32}$$

$$\left\|J(\mathbf{P}^{r+1}(t))-J(\mathbf{P}^r(t))\right\| \le \frac{\varpi_{W_1}\mathbb{T}^\beta}{\Gamma(\beta+1)}\left\|\mathbf{P}^{r+1}-\mathbf{P}^r\right\|_{K^1}. \tag{33}$$

$$\left\|J(\mathbf{F}^{r+1}(t))-J(\mathbf{F}^r(t))\right\| \le \frac{\varpi_{X_1}\mathbb{T}^\beta}{\Gamma(\beta+1)}\left\|\mathbf{F}^{r+1}-\mathbf{F}^r\right\|_{K^1}. \tag{34}$$

Where $\|\mathbf{G}^{r+1}-\mathbf{G}^r\|\to 0$, $\|\mathbf{T}^{r+1}-\mathbf{T}^r\|\to 0$, $\|\mathbf{P}^{r+1}-\mathbf{P}^r\|\to 0$, and $\|\mathbf{F}^{r+1}-\mathbf{F}^r\|\to 0$, as $r\to 0$. Hence, the operator J is continuous.

On the set of $K^1([0,\mathbb{T}],\mathfrak{D})$, we then demonstrate that the operator J is a one-to-one bounded function. For each $\mathbf{G}\in\mathscr{B}_\mathbf{G}$, $\mathbf{T}\in\mathscr{B}_\mathbf{T}$, $\mathbf{P}\in\mathscr{B}_\mathbf{P}$, $\mathbf{F}\in\mathscr{B}_\mathbf{F}$, and for $\vartheta>0$, there exist a constant $\varphi>0$ such that $\{\|J_\mathbf{G}\|,\|J_\mathbf{G}\|,\|J_\mathbf{G}\|,\|J_\mathbf{G}\|\}\le\varphi$. Also all the continuous functions on the range $t\in[0,\mathbb{T}]$ are defined as a subset of Banach space by

$$\begin{aligned}
\mathscr{B}_\mathbf{G} &= \{\mathbf{G}\in K^1([0,\mathbb{T}],\mathfrak{D}) : \|\mathbf{G}\|\le\vartheta\},\\
\mathscr{B}_\mathbf{T} &= \{\mathbf{T}\in K^1([0,\mathbb{T}],\mathfrak{D}) : \|\mathbf{T}\|\le\vartheta\},\\
\mathscr{B}_\mathbf{P} &= \{\mathbf{P}\in K^1([0,\mathbb{T}],\mathfrak{D}) : \|\mathbf{P}\|\le\vartheta\},\\
\mathscr{B}_\mathbf{F} &= \{\mathbf{F}\in K^1([0,\mathbb{T}],\mathfrak{D}) : \|\mathbf{F}\|\le\vartheta\}.
\end{aligned} \tag{35}$$

Therefore, for any $t \in [0, \mathbb{T}]$,

$$
\begin{aligned}
\|\mathbf{JG}\| &\leq \|\mathbf{G}(0)\| + \frac{1}{\Gamma(\beta)} \int_0^t (t-\nu)^{\beta-1} \|\mathrm{U}(\nu, \mathbf{G}(\nu))\| d\nu \\
&\leq \|\mathbf{G}(0)\| + \frac{\|\mathrm{U}(\nu, \mathbf{G}(\nu))\|}{\Gamma(\beta)} \int_0^t (t-\nu)^{\beta-1} d\nu \\
&\leq \|\mathbf{G}(0)\| + \varpi_{\mathrm{U}_1}(\rho + \|\mathbf{G}\|) \Big[\frac{\mathbb{T}^\beta}{\Gamma(\beta+1)}\Big] \\
&\leq \|\mathbf{G}(0)\| + \varpi_{\mathrm{U}_1}(\rho + \vartheta) \Big[\frac{\mathbb{T}^\beta}{\Gamma(\beta+1)}\Big].
\end{aligned} \tag{36}
$$

Also, we have

$$
\|\mathbf{JT}\| \leq \|\mathbf{T}(0)\| + \varpi_{\mathrm{V}_1}(\rho + \vartheta) \Big[\frac{\mathbb{T}^\beta}{\Gamma(\beta+1)}\Big]. \tag{37}
$$

$$
\|\mathbf{JP}\| \leq \|\mathbf{P}(0)\| + \varpi_{\mathrm{W}_1}(\rho + \vartheta) \Big[\frac{\mathbb{T}^\beta}{\Gamma(\beta+1)}\Big]' \tag{38}
$$

$$
\|\mathbf{JF}\| \leq \|\mathbf{F}(0)\| + \varpi_{\mathrm{X}_1}(\rho + \vartheta) \Big[\frac{\mathbb{T}^\beta}{\Gamma(\beta+1)}\Big]. \tag{39}
$$

As an alternative, consider the case where Y maps bounded sets in $\mathrm{K}^1([0, \mathbb{T}], \mathfrak{D})$ into equal continuous sets. If $0 \leq t_a \leq t_b \leq \mathbb{T}$, $\{t_a, t_b\} \in [0, \mathbb{T}]$, and $\mathbf{G} \in \mathscr{B}_{\mathbf{G}}$, $\mathbf{T} \in \mathscr{B}_{\mathbf{T}}$, $\mathbf{P} \in \mathscr{B}_{\mathbf{P}}$, $\mathbf{F} \in \mathscr{B}_{\mathbf{F}}$, then

$$
\begin{aligned}
\big\|\mathbf{JG}(t_a) - \mathbf{JG}(t_b)\big\| &= \frac{1}{\Gamma(\beta)} \Big\| \int_0^{t_a} (t_a - \nu)^{\beta-1} \mathrm{U}(\nu, \mathbf{G}(\nu)) d\nu - \int_0^{t_b} (t_b - \nu)^{\beta-1} \mathrm{U}(\nu, \mathbf{G}(\nu)) d\nu \Big\| \\
&\leq \frac{1}{\Gamma(\beta)} \Big\| \int_0^{t_a} \big[(t_a-\nu)^{\beta-1} - (t_b-\nu)^{\beta-1}\big] \mathrm{U}(\nu, \mathbf{G}(\nu)) d\nu \Big\| \\
&\quad + \frac{1}{\Gamma(\beta)} \Big\| \int_{t_a}^{t_b} (t_b - \nu)^{\beta-1} \mathrm{U}(\nu, \mathbf{G}(\nu)) d\nu \Big\| \\
&\leq \frac{\varpi_{\mathrm{U}_1}(\rho+\vartheta)}{\Gamma(\beta)} \Big\| \int_0^{t_a} \big[(t_a-\nu)^{\beta-1} - (t_b-\nu)^{\beta-1}\big] d\nu + \int_{t_a}^{t_b} (t_b-\nu)^{\beta-1} d\nu \Big\| \\
&\leq \frac{\varpi_{\mathrm{U}_1}(\rho+\vartheta)\mathbb{T}^\beta}{\Gamma(\beta+1)} \big[t_a^\beta - t_b^\beta + 2(t_b - t_a)^\beta\big].
\end{aligned} \tag{40}
$$

And

$$
\big\|\mathbf{JT}(t_a) - \mathbf{JT}(t_b)\big\| \leq \frac{\varpi_{\mathrm{V}_1}(\rho+\vartheta)\mathbb{T}^\beta}{\Gamma(\beta+1)} \big[t_a^\beta - t_b^\beta + 2(t_b - t_a)^\beta\big]. \tag{41}
$$

$$
\big\|\mathbf{JP}(t_a) - \mathbf{JP}(t_b)\big\| \leq \frac{\varpi_{\mathrm{W}_1}(\rho+\vartheta)\mathbb{T}^\beta}{\Gamma(\beta+1)} \big[t_a^\beta - t_b^\beta + 2(t_b - t_a)^\beta\big]. \tag{42}
$$

$$
\big\|\mathbf{JF}(t_a) - \mathbf{JF}(t_b)\big\| \leq \frac{\varpi_{\mathrm{X}_1}(\rho+\vartheta)\mathbb{T}^\beta}{\Gamma(\beta+1)} \big[t_a^\beta - t_b^\beta + 2(t_b - t_a)^\beta\big]. \tag{43}
$$

The above expressions approach zero when $t_a \to t_b$ on the right side of the inequality. According to the Arzela-Ascoli theorem, J is a continuous function.

Now, we prove that

$$\mathrm{Q(J)} = \{(\mathbf{G},\mathbf{T},\mathbf{P},\mathbf{F}) \in \mathrm{K}^1([0,\mathbb{T}],\mathfrak{D}) : (\mathbf{G},\mathbf{T},\mathbf{P},\mathbf{F}) = \mu(\mathbf{G},\mathbf{T},\mathbf{P},\mathbf{F})\} \tag{44}$$

is bounded for some $0 < \mu < 1$ by (1). For every $t \in [0,\mathbb{T}]$, let $(\mathbf{G},\mathbf{T},\mathbf{P},\mathbf{F}) \in \mathrm{Q(J)}$, such that $(\mathbf{G},\mathbf{T},\mathbf{P},\mathbf{F}) = \mu \mathrm{J}(\mathbf{G},\mathbf{T},\mathbf{P},\mathbf{F})$, yields

$$\begin{aligned}
\|\mathbf{G}(t)\| &\le \mathbf{G}(0) + \frac{1}{\Gamma(\beta)}\int_0^{\mathbb{T}} (t-\nu)^{\beta-1}\|\mathrm{U}(\nu,\mathbf{G}(\nu))\| d\nu \\
&\le \mathbf{G}(0) + \frac{\varpi_{\mathrm{U}_1}}{\Gamma(\beta)}\int_0^{\mathbb{T}} (t-\nu)^{\beta-1}(\rho + \|\mathbf{G}(\nu)\|) d\nu \\
&\le \mathbf{G}(0) + \frac{\rho\varpi_{\mathrm{U}_1}}{\Gamma(\beta)}\int_0^{\mathbb{T}} (t-\nu)^{\beta-1} d\nu + \frac{\varpi_{\mathrm{U}_1}}{\Gamma(\beta)}\int_0^{\mathbb{T}} (t-\nu)^{\beta-1}\|\mathbf{G}(\nu)\| d\nu \\
&\le \mathbf{G}(0) + \frac{\varpi_{\mathrm{U}_1}\mathbb{T}^\beta}{\Gamma(\beta+1)} + \frac{\varpi_{\mathrm{U}_1}\mathbb{T}^\beta}{\Gamma(\beta+1)}\int_0^{\mathbb{T}} (t-\nu)^{\beta-1}\|\mathbf{G}(\nu)\| d\nu \\
&\le \left\{\mathbf{G}(0) + \frac{\varpi_{\mathrm{U}_1}\mathbb{T}^\beta}{\Gamma(\beta+1)}\mathrm{E}_\beta(\varpi_{\mathrm{U}_1}\mathbb{T}^\beta)\right\} < \infty
\end{aligned} \tag{45}$$

And

$$\|\mathbf{T}(t)\| \le \left\{\mathbf{T}(0) + \frac{\varpi_{\mathrm{V}_1}\mathbb{T}^\beta}{\Gamma(\beta+1)}\mathrm{E}_\beta(\varpi_{\mathrm{V}_1}\mathbb{T}^\beta)\right\} < \infty. \tag{46}$$

$$\|\mathbf{P}(t)\| \le \left\{\mathbf{P}(0) + \frac{\varpi_{\mathrm{W}_1}\mathbb{T}^\beta}{\Gamma(\beta+1)}\mathrm{E}_\beta(\varpi_{\mathrm{W}_1}\mathbb{T}^\beta)\right\} < \infty. \tag{47}$$

$$\|\mathbf{F}(t)\| \le \left\{\mathbf{F}(0) + \frac{\varpi_{\mathrm{X}_1}\mathbb{T}^\beta}{\Gamma(\beta+1)}\mathrm{E}_\beta(\varpi_{\mathrm{X}_1}\mathbb{T}^\beta)\right\} < \infty. \tag{48}$$

As we have established that Q(J) is bounded, the system (1)'s solution exists because J has a fixed point which is determined by Schaefer's fixed point theorem. □

3.4. Generalized Ulam-Hyers-Rassias (UHR) stability

Utilizing the Ulam-Hyers-Rassias (UHR) Stability technique described in [38], we examine the stability of the system (1) to show that it is UHR stable.

Definition 3.1. *The proposed system (1) is generalized Ulam-Hyers-Rassias (UHR) stable with regard to* $\mathfrak{P}(t) \in \mathrm{K}^1([0,\mathbb{T}],\mathfrak{D})$ *if there exists real values* $\{\eta_\delta,\eta_\tau,\eta_\phi,\eta_\varsigma\} > 0$ *with* $\{\delta,\tau,\phi,\varsigma\} > 0$ *and for all solutions* $(\mathbf{G},\mathbf{T},\mathbf{P},\mathbf{F}) \in \mathrm{K}^1([0,\mathbb{T}],\mathfrak{D})$ *of the subsequent inequalities*

$$\begin{aligned}
\left|{}^{\mathsf{C}}D_t^\beta \mathbf{G}(t) - \mathrm{U}(t,\mathbf{G}(t))\right| &\le \mathfrak{P}(t), \\
\left|{}^{\mathsf{C}}D_t^\beta \mathbf{T}(t) - \mathrm{V}(t,\mathbf{T}(t))\right| &\le \mathfrak{P}(t), \\
\left|{}^{\mathsf{C}}D_t^\beta \mathbf{P}(t) - \mathrm{W}(t,\mathbf{P}(t))\right| &\le \mathfrak{P}(t), \\
\left|{}^{\mathsf{C}}D_t^\beta \mathbf{F}(t) - \mathrm{X}(t,\mathbf{F}(t))\right| &\le \mathfrak{P}(t),
\end{aligned} \tag{49}$$

there exists a solution $(\widetilde{\mathbf{G}},\widetilde{\mathbf{T}},\widetilde{\mathbf{P}},\widetilde{\mathbf{F}}) \in \mathrm{K}^1([0,\mathbb{T}],\mathfrak{D})$ *of proposed system (1) with*

$$\begin{aligned}
|\mathbf{G}(t) - \widetilde{\mathbf{G}}(t)| &\le \eta_\delta \mathfrak{P}(t), \\
|\mathbf{T}(t) - \widetilde{\mathbf{T}}(t)| &\le \eta_\tau \mathfrak{P}(t), \\
|\mathbf{P}(t) - \widetilde{\mathbf{P}}(t)| &\le \eta_\phi \mathfrak{P}(t), \\
|\mathbf{F}(t) - \widetilde{\mathbf{F}}(t)| &\le \eta_\varsigma \mathfrak{P}(t).
\end{aligned} \tag{50}$$

Theorem 3.5. *The proposed system (1) is generalized Ulam-Hyers-Rassias stable with respect to* $\mathrm{K}^1([0,\mathbb{T}],\mathfrak{D})$ *if*

$$(\mathbf{G},\mathbf{T},\mathbf{P},\mathbf{F})\mathbb{T}^{\beta}<1. \tag{51}$$

Proof. There exists $\mathfrak{I}=\{\delta,\tau,\phi,\varsigma\}>0$ such that

$$\int_0^t (t-v)\mathfrak{P}(v)dv \le \mathfrak{I}\mathfrak{P}(t) \tag{52}$$

is true for all $t\in[0,\mathbb{T}]$ according to definition (3.1), which designates $\mathfrak{P}$ as a non-decreasing function of t. The continuous nature of the functions $\mathbf{G}$, $\mathbf{T}$, $\mathbf{P}$, and $\mathbf{F}$ has been shown, and the Lipschitz condition is met when $(\mathbf{G},\mathbf{T},\mathbf{P},\mathbf{F})>0$. Theorem (3.3) provides a unique answer for the proposed system (1)

$$\begin{aligned}
\widetilde{\mathbf{G}}(t)&=\mathbf{G}(0)+\frac{1}{\Gamma(\beta)}\int_0^t (t-v)^{\beta-1}\|\mathrm{U}(v,\widetilde{\mathbf{G}}(v))\|dv,\\
\widetilde{\mathbf{T}}(t)&=\mathbf{T}(0)+\frac{1}{\Gamma(\beta)}\int_0^t (t-v)^{\beta-1}\|\mathrm{V}(v,\widetilde{\mathbf{T}}(v))\|dv,\\
\widetilde{\mathbf{P}}(t)&=\mathbf{P}(0)+\frac{1}{\Gamma(\beta)}\int_0^t (t-v)^{\beta-1}\|\mathrm{W}(v,\widetilde{\mathbf{P}}(v))\|dv,\\
\widetilde{\mathbf{F}}(t)&=\mathbf{F}(0)+\frac{1}{\Gamma(\beta)}\int_0^t (t-v)^{\beta-1}\|\mathrm{X}(v,\widetilde{\mathbf{F}}(v))\|dv.
\end{aligned} \tag{53}$$

When we integrate the inequalities in the definition (3.1), we acquire

$$\begin{aligned}
\left|\mathbf{G}(t)-\mathbf{G}(0)-\frac{1}{\Gamma(\beta)}\int_0^t (t-v)^{\beta-1}\mathrm{U}(v,\mathbf{G}(v))dv\right| &\le \frac{1}{\Gamma(\beta)}\int_0^t (t-v)^{\beta-1}\mathfrak{P}(v)dv \le \frac{\delta\mathfrak{P}(t)\mathbb{T}^{\beta}}{\Gamma(\beta+1)},\\
\left|\mathbf{T}(t)-\mathbf{T}(0)-\frac{1}{\Gamma(\beta)}\int_0^t (t-v)^{\beta-1}\mathrm{V}(v,\mathbf{T}(v))dv\right| &\le \frac{1}{\Gamma(\beta)}\int_0^t (t-v)^{\beta-1}\mathfrak{P}(v)dv \le \frac{\delta\mathfrak{P}(t)\mathbb{T}^{\beta}}{\Gamma(\beta+1)},\\
\left|\mathbf{P}(t)-\mathbf{P}(0)-\frac{1}{\Gamma(\beta)}\int_0^t (t-v)^{\beta-1}\mathrm{W}(v,\mathbf{P}(v))dv\right| &\le \frac{1}{\Gamma(\beta)}\int_0^t (t-v)^{\beta-1}\mathfrak{P}(v)dv \le \frac{\delta\mathfrak{P}(t)\mathbb{T}^{\beta}}{\Gamma(\beta+1)},\\
\left|\mathbf{F}(t)-\mathbf{F}(0)-\frac{1}{\Gamma(\beta)}\int_0^t (t-v)^{\beta-1}\mathrm{X}(v,\mathbf{F}(v))dv\right| &\le \frac{1}{\Gamma(\beta)}\int_0^t (t-v)^{\beta-1}\mathfrak{P}(v)dv \le \frac{\delta\mathfrak{P}(t)\mathbb{T}^{\beta}}{\Gamma(\beta+1)}.
\end{aligned} \tag{54}$$

From equation (54) and Lemma (2.2), we have

$$\begin{aligned}
\left|\mathbf{G}(t)-\widetilde{\mathbf{G}}(t)\right| &\le \left|\mathbf{G}(t)-\left[\mathbf{G}(0)+\frac{1}{\Gamma(\beta)}\int_0^t (t-v)^{\beta-1}\mathrm{U}(v,\widetilde{\mathbf{G}}(v))dv\right]\right|\\
&\le \left|\mathbf{G}(t)-\mathbf{G}(0)-\left[\frac{1}{\Gamma(\beta)}\int_0^t (t-v)^{\beta-1}\mathrm{U}(v,\widetilde{\mathbf{G}}(v))dv\right.\right.\\
&\qquad \left.\left.+\frac{1}{\Gamma(\beta)}\int_0^t (t-v)^{\beta-1}\mathrm{U}(v,\mathbf{G}(v))dv-\frac{1}{\Gamma(\beta)}\int_0^t (t-v)^{\beta-1}\mathrm{U}(v,\mathbf{G}(v))dv\right]\right|\\
&\le \left|\mathbf{G}(t)-\mathbf{G}(0)-\frac{1}{\Gamma(\beta)}\int_0^t (t-v)^{\beta-1}\mathrm{U}(v,\mathbf{G}(v))dv\right|\\
&\qquad +\frac{1}{\Gamma(\beta)}\int_0^t (t-v)^{\beta-1}\left|\mathrm{U}(v,\mathbf{G}(v))-\mathrm{U}(v,\widetilde{\mathbf{G}}(v))\right|dv\\
&\le \frac{\delta\mathfrak{P}(t)\mathbb{T}^{\beta}}{\Gamma(\beta+1)}+\frac{\varpi_{\mathrm{U}}\mathbb{T}^{\beta}}{\Gamma(\beta+1)}\int_0^t (t-v)^{\beta-1}\left|\mathbf{G}(v)-\widetilde{\mathbf{G}}(v)\right|dv \le \frac{\delta\mathfrak{P}(t)\mathbb{T}^{\beta}}{\Gamma(\beta+1)}\mathrm{E}_{\beta}(\varpi_{\mathrm{U}}\mathbb{T}^{\beta}).
\end{aligned} \tag{55}$$

$$
\begin{aligned}
\left|\mathbf{T}(t)-\widetilde{\mathbf{T}}(t)\right| &\le \left|\mathbf{T}(t)-\left[\mathbf{T}(0)+\frac{1}{\Gamma(\beta)}\int_0^t (t-\nu)^{\beta-1}\mathrm{V}(\nu,\widetilde{\mathbf{T}}(\nu))d\nu\right]\right| \\
&\le \left|\mathbf{T}(t)-\mathbf{T}(0)-\left[\frac{1}{\Gamma(\beta)}\int_0^t (t-\nu)^{\beta-1}\mathrm{V}(\nu,\widetilde{\mathbf{T}}(\nu))d\nu\right.\right. \\
&\qquad \left.\left.+\frac{1}{\Gamma(\beta)}\int_0^t (t-\nu)^{\beta-1}\mathrm{V}(\nu,\mathbf{T}(\nu))d\nu-\frac{1}{\Gamma(\beta)}\int_0^t (t-\nu)^{\beta-1}\mathrm{V}(\nu,\mathbf{T}(\nu))d\nu\right]\right| \\
&\le \left|\mathbf{T}(t)-\mathbf{T}(0)-\frac{1}{\Gamma(\beta)}\int_0^t (t-\nu)^{\beta-1}\mathrm{V}(\nu,\mathbf{T}(\nu))d\nu\right| \\
&\qquad +\frac{1}{\Gamma(\beta)}\int_0^t (t-\nu)^{\beta-1}\left|\mathrm{V}(\nu,\mathbf{T}(\nu))-\mathrm{V}(\nu,\widetilde{\mathbf{T}}(\nu))\right|d\nu \\
&\le \frac{\tau\mathfrak{P}(t)\mathbb{T}^\beta}{\Gamma(\beta+1)}+\frac{\varpi_{\mathrm{V}}\mathbb{T}^\beta}{\Gamma(\beta+1)}\int_0^t (t-\nu)^{\beta-1}\left|\mathbf{T}(\nu)-\widetilde{\mathbf{T}}(\nu)\right|d\nu \le \frac{\tau\mathfrak{P}(t)\mathbb{T}^\beta}{\Gamma(\beta+1)}\mathrm{E}_\beta(\varpi_{\mathrm{V}}\mathbb{T}^\beta).
\end{aligned}
\tag{56}
$$

$$
\begin{aligned}
\left|\mathbf{P}(t)-\widetilde{\mathbf{P}}(t)\right| &\le \left|\mathbf{P}(t)-\left[\mathbf{P}(0)+\frac{1}{\Gamma(\beta)}\int_0^t (t-\nu)^{\beta-1}\mathrm{W}(\nu,\widetilde{\mathbf{P}}(\nu))d\nu\right]\right| \\
&\le \left|\mathbf{P}(t)-\mathbf{P}(0)-\left[\frac{1}{\Gamma(\beta)}\int_0^t (t-\nu)^{\beta-1}\mathrm{W}(\nu,\widetilde{\mathbf{P}}(\nu))d\nu\right.\right. \\
&\qquad \left.\left.+\frac{1}{\Gamma(\beta)}\int_0^t (t-\nu)^{\beta-1}\mathrm{W}(\nu,\mathbf{P}(\nu))d\nu-\frac{1}{\Gamma(\beta)}\int_0^t (t-\nu)^{\beta-1}\mathrm{W}(\nu,\mathbf{P}(\nu))d\nu\right]\right| \\
&\le \left|\mathbf{P}(t)-\mathbf{P}(0)-\frac{1}{\Gamma(\beta)}\int_0^t (t-\nu)^{\beta-1}\mathrm{W}(\nu,\mathbf{P}(\nu))d\nu\right| \\
&\qquad +\frac{1}{\Gamma(\beta)}\int_0^t (t-\nu)^{\beta-1}\left|\mathrm{W}(\nu,\mathbf{P}(\nu))-\mathrm{W}(\nu,\widetilde{\mathbf{P}}(\nu))\right|d\nu \\
&\le \frac{\phi\mathfrak{P}(t)\mathbb{T}^\beta}{\Gamma(\beta+1)}+\frac{\varpi_{\mathrm{W}}\mathbb{T}^\beta}{\Gamma(\beta+1)}\int_0^t (t-\nu)^{\beta-1}\left|\mathbf{P}(\nu)-\widetilde{\mathbf{P}}(\nu)\right|d\nu \le \frac{\phi\mathfrak{P}(t)\mathbb{T}^\beta}{\Gamma(\beta+1)}\mathrm{E}_\beta(\varpi_{\mathrm{W}}\mathbb{T}^\beta).
\end{aligned}
\tag{57}
$$

$$
\begin{aligned}
\left|\mathbf{F}(t)-\widetilde{\mathbf{F}}(t)\right| &\le \left|\mathbf{F}(t)-\left[\mathbf{F}(0)+\frac{1}{\Gamma(\beta)}\int_0^t (t-\nu)^{\beta-1}\mathrm{X}(\nu,\widetilde{\mathbf{F}}(\nu))d\nu\right]\right| \\
&\le \left|\mathbf{F}(t)-\mathbf{F}(0)-\left[\frac{1}{\Gamma(\beta)}\int_0^t (t-\nu)^{\beta-1}\mathrm{X}(\nu,\widetilde{\mathbf{F}}(\nu))d\nu\right.\right. \\
&\qquad \left.\left.+\frac{1}{\Gamma(\beta)}\int_0^t (t-\nu)^{\beta-1}\mathrm{X}(\nu,\mathbf{F}(\nu))d\nu-\frac{1}{\Gamma(\beta)}\int_0^t (t-\nu)^{\beta-1}\mathrm{X}(\nu,\mathbf{F}(\nu))d\nu\right]\right| \\
&\le \left|\mathbf{F}(t)-\mathbf{F}(0)-\frac{1}{\Gamma(\beta)}\int_0^t (t-\nu)^{\beta-1}\mathrm{X}(\nu,\mathbf{F}(\nu))d\nu\right| \\
&\qquad +\frac{1}{\Gamma(\beta)}\int_0^t (t-\nu)^{\beta-1}\left|\mathrm{X}(\nu,\mathbf{F}(\nu))-\mathrm{X}(\nu,\widetilde{\mathbf{F}}(\nu))\right|d\nu \\
&\le \frac{\varsigma\mathfrak{P}(t)\mathbb{T}^\beta}{\Gamma(\beta+1)}+\frac{\varpi_{\mathrm{X}}\mathbb{T}^\beta}{\Gamma(\beta+1)}\int_0^t (t-\nu)^{\beta-1}\left|\mathbf{F}(\nu)-\widetilde{\mathbf{F}}(\nu)\right|d\nu \le \frac{\varsigma\mathfrak{P}(t)\mathbb{T}^\beta}{\Gamma(\beta+1)}\mathrm{E}_\beta(\varpi_{\mathrm{X}}\mathbb{T}^\beta).
\end{aligned}
\tag{58}
$$

Let $\frac{\delta\mathfrak{P}(t)\mathbb{T}^\beta}{\Gamma(\beta+1)}\mathrm{E}_\beta(\varpi_{\mathrm{U}}\mathbb{T}^\beta)=\chi_\delta$, $\frac{\tau\mathfrak{P}(t)\mathbb{T}^\beta}{\Gamma(\beta+1)}\mathrm{E}_\beta(\varpi_{\mathrm{V}}\mathbb{T}^\beta)=\chi_\tau$, $\frac{\phi\mathfrak{P}(t)\mathbb{T}^\beta}{\Gamma(\beta+1)}\mathrm{E}_\beta(\varpi_{\mathrm{W}}\mathbb{T}^\beta)=\chi_\phi$, and

$\frac{\varsigma\mathfrak{P}(t)\mathbb{T}^{\beta}}{\Gamma(\beta+1)}\mathrm{E}_{\beta}(\varpi_{\mathrm{X}}\mathbb{T}^{\beta}) = \chi_{\varsigma}$, then we have

$$\begin{aligned}
|\mathbf{G}(t)-\widetilde{\mathbf{G}}(t)| &\leq \chi_{\delta}\mathfrak{P}(t),\\
|\mathbf{T}(t)-\widetilde{\mathbf{T}}(t)| &\leq \chi_{\tau}\mathfrak{P}(t),\\
|\mathbf{P}(t)-\widetilde{\mathbf{P}}(t)| &\leq \chi_{\phi}\mathfrak{P}(t),\\
|\mathbf{F}(t)-\widetilde{\mathbf{F}}(t)| &\leq \chi_{\varsigma}\mathfrak{P}(t).
\end{aligned} \tag{59}$$

□

4. Numerical scheme

In the literature, it has been proposed that the power-law kernel-based Caputo derivative is suitable for mimicking power-law processes in practical problems. We use a numerical scheme based on a Newton polynomial to solve the system (1) numerically.

$$\begin{aligned}
{}_0^{\mathrm{C}}D_t^{\beta}\mathbf{G}(t) &= g_1\mathbf{G}+\gamma_1\mathbf{GF}-\gamma_2\mathbf{GP}+\gamma_3\mathbf{T},\\
{}_0^{\mathrm{C}}D_t^{\beta}\mathbf{T}(t) &= g_2\mathbf{T}+\lambda_1\mathbf{GT}-\lambda_2\mathbf{PT},\\
{}_0^{\mathrm{C}}D_t^{\beta}\mathbf{P}(t) &= g_3\mathbf{P}\big(1-\frac{\mathbf{P}}{Q_1}\big)+\frac{\omega_1\mathbf{P}}{\alpha+\mathbf{G}}-\omega_2\mathbf{PT}-\omega_3\mathbf{FP}-\omega_4\mathbf{GP},\\
{}_0^{\mathrm{C}}D_t^{\beta}\mathbf{F}(t) &= g_4\mathbf{F}\big(1-\frac{\mathbf{F}}{Q_2}\big)+\xi_1\mathbf{FP}-\frac{\xi_2\mathbf{F}}{\alpha+\mathbf{G}}-\xi_3\mathbf{TF}.
\end{aligned} \tag{60}$$

We'll write the aforementioned system in the following way to make it easier to use:

$$\begin{aligned}
\mathbb{K}_1(t,\mathbf{G},\mathbf{T},\mathbf{P},\mathbf{F}) &= g_1\mathbf{G}+\gamma_1\mathbf{GF}-\gamma_2\mathbf{GP}+\gamma_3\mathbf{T},\\
\mathbb{K}_1(t,\mathbf{G},\mathbf{T},\mathbf{P},\mathbf{F}) &= g_2\mathbf{T}+\lambda_1\mathbf{GT}-\lambda_2\mathbf{PT},\\
\mathbb{K}_1(t,\mathbf{G},\mathbf{T},\mathbf{P},\mathbf{F}) &= g_3\mathbf{P}\big(1-\frac{\mathbf{P}}{Q_1}\big)+\frac{\omega_1\mathbf{P}}{\alpha+\mathbf{G}}-\omega_2\mathbf{PT}-\omega_3\mathbf{FP}-\omega_4\mathbf{GP},\\
\mathbb{K}_1(t,\mathbf{G},\mathbf{T},\mathbf{P},\mathbf{F}) &= g_4\mathbf{F}\big(1-\frac{\mathbf{F}}{Q_2}\big)+\xi_1\mathbf{FP}-\frac{\xi_2\mathbf{F}}{\alpha+\mathbf{G}}-\xi_3\mathbf{TF}.
\end{aligned} \tag{61}$$

We obtain the following after using fractional integral:

$$\mathbf{G}(t_w+1) = \mathbf{G}(0)+\frac{1}{\Gamma(\beta)}\sum_{q=2}^{w}\int_{t_q}^{t_{q+1}}\mathbb{K}_1(t,\mathbf{G},\mathbf{T},\mathbf{P},\mathbf{F})(t_{w+1}-\nu)^{\beta-1}d\nu, \tag{62}$$

$$\mathbf{T}(t_w+1) = \mathbf{T}(0)+\frac{1}{\Gamma(\beta)}\sum_{q=2}^{w}\int_{t_q}^{t_{q+1}}\mathbb{K}_2(t,\mathbf{G},\mathbf{T},\mathbf{P},\mathbf{F})(t_{w+1}-\nu)^{\beta-1}d\nu, \tag{63}$$

$$\mathbf{P}(t_w+1) = \mathbf{P}(0)+\frac{1}{\Gamma(\beta)}\sum_{q=2}^{w}\int_{t_q}^{t_{q+1}}\mathbb{K}_3(t,\mathbf{G},\mathbf{T},\mathbf{P},\mathbf{F})(t_{w+1}-\nu)^{\beta-1}d\nu, \tag{64}$$

$$\mathbf{F}(t_w+1) = \mathbf{F}(0)+\frac{1}{\Gamma(\beta)}\sum_{q=2}^{w}\int_{t_q}^{t_{q+1}}\mathbb{K}_4(t,\mathbf{G},\mathbf{T},\mathbf{P},\mathbf{F})(t_{w+1}-\nu)^{\beta-1}d\nu, \tag{65}$$

We will now review the Newton polynomial:

$$\begin{aligned}
\mathsf{P}(t,\mathbf{G},\mathbf{T},\mathbf{P},\mathbf{F}) \simeq\ & \mathsf{P}(t_{w-2},\mathbf{G}^{w-2},\mathbf{T}^{w-2},\mathbf{P}^{w-2},\mathbf{F}^{w-2}) \\
& + \frac{1}{\Delta t}\Big\{\mathsf{P}(t_{w-1},\mathbf{G}^{w-1},\mathbf{T}^{w-1},\mathbf{P}^{w-1},\mathbf{F}^{w-1}) - \mathsf{P}(t_{w-2},\mathbf{G}^{w-2},\mathbf{T}^{w-2},\mathbf{P}^{w-2},\mathbf{F}^{w-2})\Big\} \\
& \times (\nu - t_{w-2}) \\
& + \frac{1}{2\Delta t^2}\Big\{\mathsf{P}(t_w,\mathbf{G}^{w},\mathbf{T}^{w},\mathbf{P}^{w},\mathbf{F}^{w}) - 2\mathsf{P}(t_{w-2},\mathbf{G}^{w-1},\mathbf{T}^{w-1},\mathbf{P}^{w-1},\mathbf{F}^{w-1}) \\
& + \mathsf{P}(t_{w-2},\mathbf{G}^{w-2},\mathbf{T}^{w-2},\mathbf{P}^{w-2},\mathbf{F}^{w-2})\Big\} \times (\nu - t_{w-2})(\nu - t_{w-1})
\end{aligned} \tag{66}$$

Replacing the Newton polynomial (66) into equation (62)-(65), we have

$$\begin{aligned}
\mathbf{G}_{(w+1)} =\ & \mathbf{G}(0) + \frac{1}{\Gamma(\beta)}\sum_{q=2}^{w}\mathbb{K}_1\left(t_{q-2},\mathbf{G}^{q-2},\mathbf{T}^{q-2},\mathbf{P}^{q-2},\mathbf{F}^{q-2}\right) \times \int_{t_q}^{t_{q+1}}(t_{w+1}-\nu)^{\beta-1}d\nu \\
& + \frac{1}{\Gamma(\beta)}\sum_{q=2}^{w}\frac{1}{\Delta t}\Big\{\mathbb{K}_1\left(t_{q-1},\mathbf{G}^{q-1},\mathbf{T}^{q-1},\mathbf{P}^{q-1},\mathbf{F}^{q-1}\right) - \mathbb{K}_1\left(t_{q-2},\mathbf{G}^{q-2},\mathbf{T}^{q-2},\mathbf{P}^{q-2},\mathbf{F}^{q-2}\right)\Big\} \\
& \times \int_{t_q}^{t_{q+1}}(\nu - t_{q-2})(t_{w+1}-\nu)^{\beta-1}d\nu \\
& + \frac{1}{\Gamma(\beta)}\sum_{q=2}^{w}\frac{1}{2\Delta t^2}\Big\{\mathbb{K}_1\left(t_{q},\mathbf{G}^{q},\mathbf{T}^{q},\mathbf{P}^{q},\mathbf{F}^{q}\right) - 2\mathbb{K}_1\left(t_{q-1},\mathbf{G}^{q-1},\mathbf{T}^{q-1},\mathbf{P}^{q-1},\mathbf{F}^{q-1}\right) \\
& + \mathbb{K}_1\left(t_{q-2},\mathbf{G}^{q-2},\mathbf{T}^{q-2},\mathbf{P}^{q-2},\mathbf{F}^{q-2}\right)\Big\} \times \int_{t_q}^{t_{q+1}}(\nu - t_{q-2})(\nu - t_{q-1})(t_{w+1}-\nu)^{\beta-1}d\nu
\end{aligned} \tag{67}$$

$$\begin{aligned}
\mathbf{T}_{(w+1)} =\ & \mathbf{T}(0) + \frac{1}{\Gamma(\beta)}\sum_{q=2}^{w}\mathbb{K}_2\left(t_{q-2},\mathbf{G}^{q-2},\mathbf{T}^{q-2},\mathbf{P}^{q-2},\mathbf{F}^{q-2}\right) \times \int_{t_q}^{t_{q+1}}(t_{w+1}-\nu)^{\beta-1}d\nu \\
& + \frac{1}{\Gamma(\beta)}\sum_{q=2}^{w}\frac{1}{\Delta t}\Big\{\mathbb{K}_2\left(t_{q-1},\mathbf{G}^{q-1},\mathbf{T}^{q-1},\mathbf{P}^{q-1},\mathbf{F}^{q-1}\right) - \mathbb{K}_2\left(t_{q-2},\mathbf{G}^{q-2},\mathbf{T}^{q-2},\mathbf{P}^{q-2},\mathbf{F}^{q-2}\right)\Big\} \\
& \times \int_{t_q}^{t_{q+1}}(\nu - t_{q-2})(t_{w+1}-\nu)^{\beta-1}d\nu \\
& + \frac{1}{\Gamma(\beta)}\sum_{q=2}^{w}\frac{1}{2\Delta t^2}\Big\{\mathbb{K}_2\left(t_{q},\mathbf{G}^{q},\mathbf{T}^{q},\mathbf{P}^{q},\mathbf{F}^{q}\right) - 2\mathbb{K}_2\left(t_{q-1},\mathbf{G}^{q-1},\mathbf{T}^{q-1},\mathbf{P}^{q-1},\mathbf{F}^{q-1}\right) \\
& + \mathbb{K}_2\left(t_{q-2},\mathbf{G}^{q-2},\mathbf{T}^{q-2},\mathbf{P}^{q-2},\mathbf{F}^{q-2}\right)\Big\} \times \int_{t_q}^{t_{q+1}}(\nu - t_{q-2})(\nu - t_{q-1})(t_{w+1}-\nu)^{\beta-1}d\nu
\end{aligned} \tag{68}$$

$$\begin{aligned}
\mathbf{P}_{(w+1)} =\ & \mathbf{P}(0) + \frac{1}{\Gamma(\beta)}\sum_{q=2}^{w}\mathbb{K}_3\left(t_{q-2},\mathbf{G}^{q-2},\mathbf{T}^{q-2},\mathbf{P}^{q-2},\mathbf{F}^{q-2}\right) \times \int_{t_q}^{t_{q+1}}(t_{w+1}-\nu)^{\beta-1}d\nu \\
& + \frac{1}{\Gamma(\beta)}\sum_{q=2}^{w}\frac{1}{\Delta t}\Big\{\mathbb{K}_3\left(t_{q-1},\mathbf{G}^{q-1},\mathbf{T}^{q-1},\mathbf{P}^{q-1},\mathbf{F}^{q-1}\right) - \mathbb{K}_3\left(t_{q-2},\mathbf{G}^{q-2},\mathbf{T}^{q-2},\mathbf{P}^{q-2},\mathbf{F}^{q-2}\right)\Big\} \\
& \times \int_{t_q}^{t_{q+1}}(\nu - t_{q-2})(t_{w+1}-\nu)^{\beta-1}d\nu \\
& + \frac{1}{\Gamma(\beta)}\sum_{q=2}^{w}\frac{1}{2\Delta t^2}\Big\{\mathbb{K}_3\left(t_{q},\mathbf{G}^{q},\mathbf{T}^{q},\mathbf{P}^{q},\mathbf{F}^{q}\right) - 2\mathbb{K}_3\left(t_{q-1},\mathbf{G}^{q-1},\mathbf{T}^{q-1},\mathbf{P}^{q-1},\mathbf{F}^{q-1}\right) \\
& + \mathbb{K}_3\left(t_{q-2},\mathbf{G}^{q-2},\mathbf{T}^{q-2},\mathbf{P}^{q-2},\mathbf{F}^{q-2}\right)\Big\} \times \int_{t_q}^{t_{q+1}}(\nu - t_{q-2})(\nu - t_{q-1})(t_{w+1}-\nu)^{\beta-1}d\nu
\end{aligned} \tag{69}$$

$$
\begin{aligned}
\mathbf{F}_{(w+1)} &= \mathbf{F}(0) + \frac{1}{\Gamma(\beta)} \sum_{q=2}^{w} \mathbb{K}_4\left(t_{q-2}, \mathbf{G}^{q-2}, \mathbf{T}^{q-2}, \mathbf{P}^{q-2}, \mathbf{F}^{q-2}\right) \times \int_{t_q}^{t_{q+1}} (t_{w+1} - \nu)^{\beta-1} d\nu \\
&+ \frac{1}{\Gamma(\beta)} \sum_{q=2}^{w} \frac{1}{\Delta t} \Big\{ \mathbb{K}_4\left(t_{q-1}, \mathbf{G}^{q-1}, \mathbf{T}^{q-1}, \mathbf{P}^{q-1}, \mathbf{F}^{q-1}\right) - \mathbb{K}_4\left(t_{q-2}, \mathbf{G}^{q-2}, \mathbf{T}^{q-2}, \mathbf{P}^{q-2}, \mathbf{F}^{q-2}\right) \Big\} \\
&\times \int_{t_q}^{t_{q+1}} (\nu - t_{q-2})(t_{w+1} - \nu)^{\beta-1} d\nu \\
&+ \frac{1}{\Gamma(\beta)} \sum_{q=2}^{w} \frac{1}{2\Delta t^2} \Big\{ \mathbb{K}_4\left(t_q, \mathbf{G}^q, \mathbf{T}^q, \mathbf{P}^q, \mathbf{F}^q\right) - 2\mathbb{K}_4\left(t_{q-1}, \mathbf{G}^{q-1}, \mathbf{T}^{q-1}, \mathbf{P}^{q-1}, \mathbf{F}^{q-1}\right) \\
&+ \mathbb{K}_4\left(t_{q-2}, \mathbf{G}^{q-2}, \mathbf{T}^{q-2}, \mathbf{P}^{q-2}, \mathbf{F}^{q-2}\right) \Big\} \times \int_{t_q}^{t_{q+1}} (\nu - t_{q-2})(\nu - t_{q-1})(t_{w+1} - \nu)^{\beta-1} d\nu
\end{aligned} \tag{70}
$$

The integral indicated in the equations above can be calculated using the formulas below.

$$
\int_{t_q}^{t_{q+1}} (t_{w+1} - \nu)^{\beta-1} d\nu = \frac{(\Delta t)^{\beta}}{\beta} \Big[(w-q+1)^{\beta} - (w-q)^{\beta} \Big] \tag{71}
$$

$$
\int_{t_q}^{t_{q+1}} (\nu - t_{q-2})(t_{w+1} - \nu)^{\beta-1} d\nu = \frac{(\Delta t)^{\beta+1}}{\beta(\beta+1)} \Big[(w-q+1)^{\beta}(w-q+3+2\beta) - (w-q)^{\beta}(w-q+3+3\beta) \Big] \tag{72}
$$

$$
\begin{aligned}
&\int_{t_q}^{t_{q+1}} (\nu - t_{q-2})(\nu - t_{q-1})(t_{w+1} - \nu)^{\beta-1} d\nu \\
&\quad = \frac{(\Delta t)^{\beta+2}}{\beta(\beta+1)(\beta+2)} \times \Big[(w-q+1)^{\beta} \Big\{ 2(w-q)^2 + (3\beta+10)(w-q) + 2\beta^2 + 9\beta + 12 \Big\} \\
&\quad - (w-q)^{\beta} \Big\{ 2(w-q)^2 + (5\beta+10)(w-q) + 6\beta^2 + 18\beta + 12 \Big\} \Big]
\end{aligned} \tag{73}
$$

Hence, we get finally

$$
\begin{aligned}
\mathbf{G}(t_{w+1}) &= \mathbf{G}(0) + \frac{(\Delta t)^{\beta}}{\Gamma(\beta+1)} \sum_{q=2}^{w} \mathbb{K}_1(t_{q-2}, \mathbf{G}^{q-2}, \mathbf{T}^{q-2}, \mathbf{P}^{q-2}, \mathbf{F}^{q-2}) \times \mathfrak{Y}_1 \\
&+ \frac{(\Delta t)^{\beta}}{\Gamma(\beta+2)} \sum_{q=2}^{w} \Big[\mathbb{K}_1(t_{q-1}, \mathbf{G}^{q-1}, \mathbf{T}^{q-1}, \mathbf{P}^{q-1}, \mathbf{F}^{q-1}) - \mathbb{K}_1(t_{q-2}, \mathbf{G}^{q-2}, \mathbf{T}^{q-2}, \mathbf{P}^{q-2}, \mathbf{F}^{q-2}) \Big] \times \mathfrak{Y}_2 \\
&+ \frac{\beta(\Delta t)^{\beta}}{2\Gamma(\beta+3)} \sum_{q=2}^{w} \Big[\mathbb{K}_1(t_q, \mathbf{G}^q, \mathbf{T}^q, \mathbf{P}^q, \mathbf{F}^q) - 2\mathbb{K}_1(t_{q-1}, \mathbf{G}^{q-1}, \mathbf{T}^{q-1}, \mathbf{P}^{q-1}, \mathbf{F}^{q-1}) \\
&+ \mathbb{K}_1(t_{q-2}, \mathbf{G}^{q-2}, \mathbf{T}^{q-2}, \mathbf{P}^{q-2}, \mathbf{F}^{q-2}) \Big] \times \mathfrak{Y}_3.
\end{aligned} \tag{74}
$$

Where,

$$
\begin{aligned}
\mathfrak{Y}_1 &= (w-q+1)^{\beta} - (w-q)^{\beta} \\
\mathfrak{Y}_2 &= (w-q+1)^{\beta}(w-q+3+2\beta) - (w-q)^{\beta}(w-q+3+3\beta) \\
\mathfrak{Y}_3 &= (w-q+1)^{\beta} \Big[2(w-q)^2 + (3\beta+10)(w-q) + 2\beta^2 + 9\beta + 12 \Big] \\
&\quad -(w-q)^{\beta} \Big[2(w-q)^2 + (5\beta+10)(w-q) + 6\beta^2 + 18\beta + 12 \Big].
\end{aligned} \tag{75}
$$

Similarly, we get

$$\begin{aligned}\mathbf{T}(t_{w+1}) &= \mathbf{T}(0) + \frac{(\Delta t)^\beta}{\Gamma(\beta+1)} \sum_{q=2}^{w} \mathbb{K}_2(t_{q-2}, \mathbf{G}^{q-2}, \mathbf{T}^{q-2}, \mathbf{P}^{q-2}, \mathbf{F}^{q-2}) \times \mathfrak{Y}_1 \\ &+ \frac{(\Delta t)^\beta}{\Gamma(\beta+2)} \sum_{q=2}^{w} \Big[\mathbb{K}_2(t_{q-1}, \mathbf{G}^{q-1}, \mathbf{T}^{q-1}, \mathbf{P}^{q-1}, \mathbf{F}^{q-1}) - \mathbb{K}_2(t_{q-2}, \mathbf{G}^{q-2}, \mathbf{T}^{q-2}, \mathbf{P}^{q-2}, \mathbf{F}^{q-2})\Big] \times \mathfrak{Y}_2 \\ &+ \frac{\beta(\Delta t)^\beta}{2\Gamma(\beta+3)} \sum_{q=2}^{w} \Big[\mathbb{K}_2(t_q, \mathbf{G}^q, \mathbf{T}^q, \mathbf{P}^q, \mathbf{F}^q) - 2\mathbb{K}_2(t_{q-1}, \mathbf{G}^{q-1}, \mathbf{T}^{q-1}, \mathbf{P}^{q-1}, \mathbf{F}^{q-1}) \\ &+ \mathbb{K}_2(t_{q-2}, \mathbf{G}^{q-2}, \mathbf{T}^{q-2}, \mathbf{P}^{q-2}, \mathbf{F}^{q-2})\Big] \times \mathfrak{Y}_3.\end{aligned} \tag{76}$$

$$\begin{aligned}\mathbf{P}(t_{w+1}) &= \mathbf{P}(0) + \frac{(\Delta t)^\beta}{\Gamma(\beta+1)} \sum_{q=2}^{w} \mathbb{K}_3(t_{q-2}, \mathbf{G}^{q-2}, \mathbf{T}^{q-2}, \mathbf{P}^{q-2}, \mathbf{F}^{q-2}) \times \mathfrak{Y}_1 \\ &+ \frac{(\Delta t)^\beta}{\Gamma(\beta+2)} \sum_{q=2}^{w} \Big[\mathbb{K}_3(t_{q-1}, \mathbf{G}^{q-1}, \mathbf{T}^{q-1}, \mathbf{P}^{q-1}, \mathbf{F}^{q-1}) - \mathbb{K}_3(t_{q-2}, \mathbf{G}^{q-2}, \mathbf{T}^{q-2}, \mathbf{P}^{q-2}, \mathbf{F}^{q-2})\Big] \times \mathfrak{Y}_2 \\ &+ \frac{\beta(\Delta t)^\beta}{2\Gamma(\beta+3)} \sum_{q=2}^{w} \Big[\mathbb{K}_3(t_q, \mathbf{G}^q, \mathbf{T}^q, \mathbf{P}^q, \mathbf{F}^q) - 2\mathbb{K}_3(t_{q-1}, \mathbf{G}^{q-1}, \mathbf{T}^{q-1}, \mathbf{P}^{q-1}, \mathbf{F}^{q-1}) \\ &+ \mathbb{K}_3(t_{q-2}, \mathbf{G}^{q-2}, \mathbf{T}^{q-2}, \mathbf{P}^{q-2}, \mathbf{F}^{q-2})\Big] \times \mathfrak{Y}_3.\end{aligned} \tag{77}$$

$$\begin{aligned}\mathbf{F}(t_{w+1}) &= \mathbf{F}(0) + \frac{(\Delta t)^\beta}{\Gamma(\beta+1)} \sum_{q=2}^{w} \mathbb{K}_4(t_{q-2}, \mathbf{G}^{q-2}, \mathbf{T}^{q-2}, \mathbf{P}^{q-2}, \mathbf{F}^{q-2}) \times \mathfrak{Y}_1 \\ &+ \frac{(\Delta t)^\beta}{\Gamma(\beta+2)} \sum_{q=2}^{w} \Big[\mathbb{K}_4(t_{q-1}, \mathbf{G}^{q-1}, \mathbf{T}^{q-1}, \mathbf{P}^{q-1}, \mathbf{F}^{q-1}) - \mathbb{K}_4(t_{q-2}, \mathbf{G}^{q-2}, \mathbf{T}^{q-2}, \mathbf{P}^{q-2}, \mathbf{F}^{q-2})\Big] \times \mathfrak{Y}_2 \\ &+ \frac{\beta(\Delta t)^\beta}{2\Gamma(\beta+3)} \sum_{q=2}^{w} \Big[\mathbb{K}_4(t_q, \mathbf{G}^q, \mathbf{T}^q, \mathbf{P}^q, \mathbf{F}^q) - 2\mathbb{K}_4(t_{q-1}, \mathbf{G}^{q-1}, \mathbf{T}^{q-1}, \mathbf{P}^{q-1}, \mathbf{F}^{q-1}) \\ &+ \mathbb{K}_4(t_{q-2}, \mathbf{G}^{q-2}, \mathbf{T}^{q-2}, \mathbf{P}^{q-2}, \mathbf{F}^{q-2})\Big] \times \mathfrak{Y}_3.\end{aligned} \tag{78}$$

5. Numerical Simulation

The model's numerical simulations have been carried out using the generalized two-step Lagrange polynomial for the power law kernel and the parametric values from [5] which are: $\delta_1 = 0.00095$, $\delta_2 = 0.0099$, $\delta_3 = 0.00025$, $\delta_4 = \frac{0.0002}{1000}$, $\gamma_1 = 0.0029$, $\gamma_2 = 0.00099$, $\gamma_3 = 1.0$, $\lambda_1 = 0.00025$, $\lambda_2 = 0.00565$, $\omega_1 = 0.00108$, $\omega_2 = 0.00001$, $\omega_3 = 0.0031$, $\omega_4 = 10.1$, $\xi_1 = \frac{175}{1000}$, $\xi_2 = \frac{190}{1000}$, $\xi_3 = \frac{61}{1000}$, $\alpha = 0.01$, $Q_1 = 1000{,}000$, and $Q_2 = 10{,}000$. We have used the value of $\Delta(t) = 0.01$. While the starting values of the continually changing species are $\mathbf{G}(0) = 0.04$, $\mathbf{T}(0) = 0.07$, $\mathbf{P}(0) = 17.5$, and $\mathbf{F}(0) = 7.8$. The simulations' main objectives are to confirm the analytical findings of this work and to clarify the dynamic behaviors of the organisms under consideration, particularly the plankton and fish populations in marine ecosystems under accelerated global warming. By dispersing energy, heat, and materials, marine ecosystems significantly contribute to restoring the equilibrium of the environment. Simulations of the proposed design model show that when analyzing internal behavior, the overall density of

all the segments will fluctuate between 0 and 1. Analytical outcomes and to describe the model's application methodology examined the negative effects of the frequent, fast environmental concentration on marine ecosystems in figures (1)-(4) at fractional orders $\alpha = 1.0, 0.95, 0.85, 0.80$. Also, the impact of fractional order at different values $\alpha = 0.65, 0.60, 0.55, 0.50$ is shown in figures (5)-(8) to observe the complete transmission by changing values. The quantity of greenhouse gases in the natural environment is constantly rising, and this means that the atmospheric temperature is rising proportionately to the quick volume of GGs. This quick volume of GGs also leads to the introduction of acidification in ocean water, that eliminates both plankton diversity and fisheries resources in oceans. In addition, marine fisheries resources are in danger and reducing primarily as a consequence of fast global warming; however, fish populations decrease proportionately with a reduction in plankton diversity due to shortages of food. Thus, the growing amount of greenhouse gases encourages global warming, which drastically lowers the planktonic population through increased acidity and warming, and the associated outcomes are significantly reduced. A comparison of the results is drawn in figures (9)-(12) and (13)-(16) by using power law, exponential law, and Mittag Leffler kernel at fractal dimension $\beta = 0.9$ and $\beta = 0.8$ respectively and solution bounded to the steady state point rapidly. Simulation of all compartments in feasible regions with chaotic form and bounded regions at different fractal fractional values is shown in figures (17)-(19). Fractional-order derivations are more effective than traditional integer-order models in explaining physical processes. The present study examines the extent to which global warming will affect plankton and fish populations in marine bio-diversity, taking into account the impact of fractional memory. Additionally, the results at different fractal dimensions and bounded solitude in the domain shown in figures (17)-(19) are discussed, providing support for both theoretical and experimental observations. Additionally, this study forecasts the future of marine ecosystems, including fish and plankton populations, as well as rapid global warming through long-term numerical analysis of the dynamic behavior of dynamic organisms.

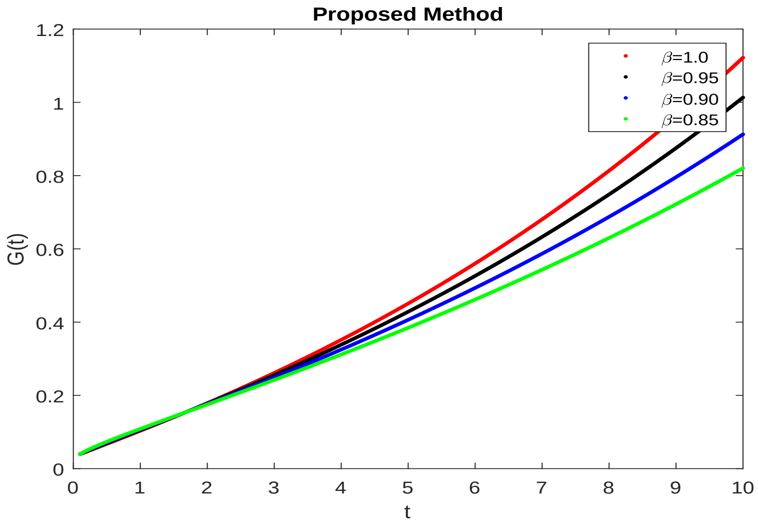

Figure 1: Simulation of $\mathbf{G}(t)$ with Caputo fractional operator

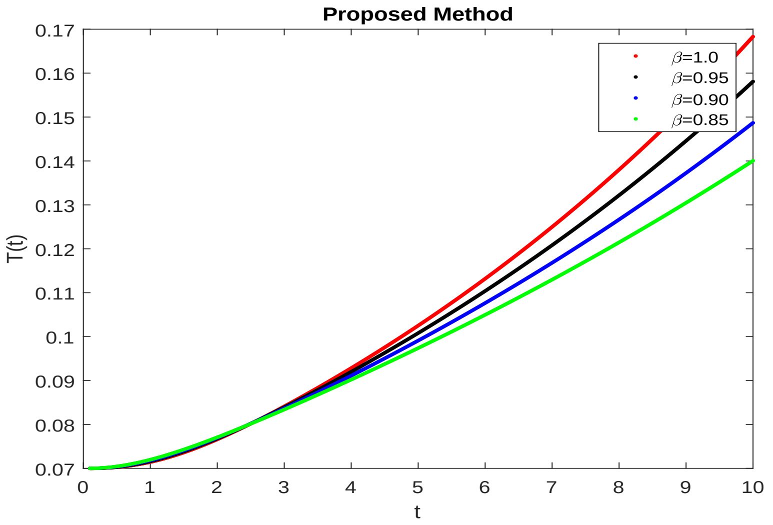

Figure 2: Simulation of $\mathbf{T}(t)$ with Caputo fractional operator

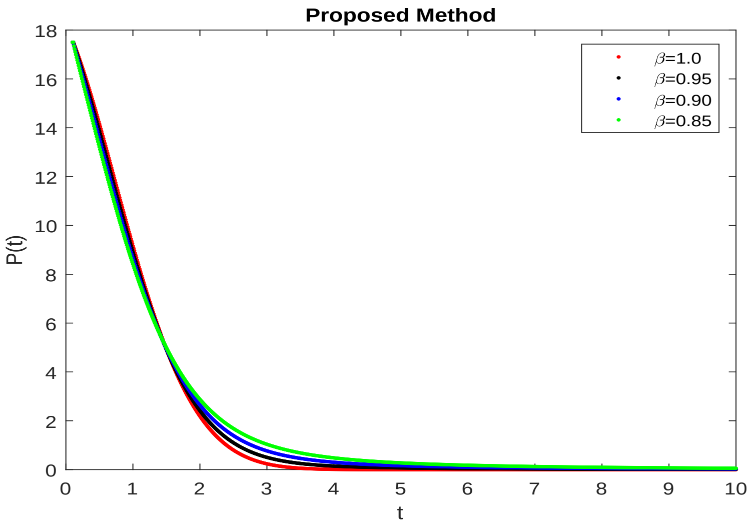

Figure 3: Simulation of $\mathbf{P}(t)$ with Caputo fractional operator

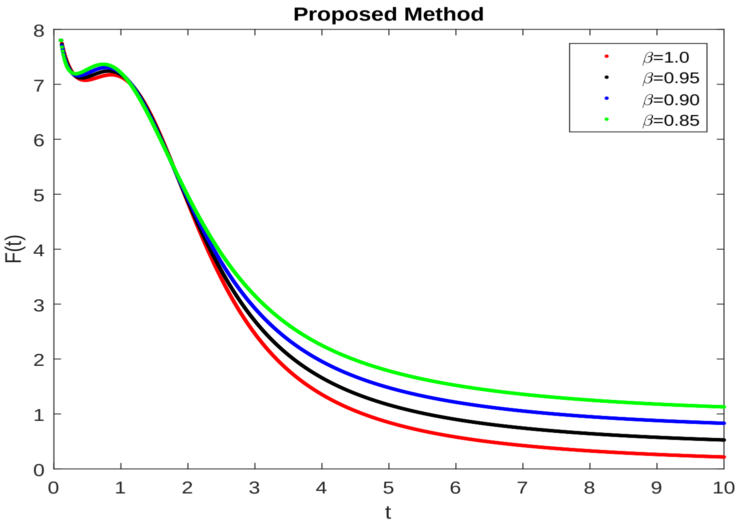

Figure 4: Simulation of $\mathbf{F}(t)$ with Caputo fractional operator

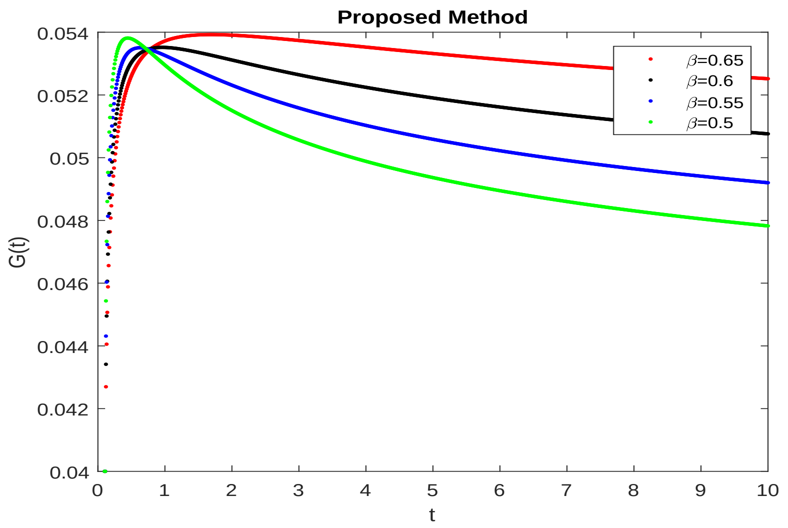

Figure 5: Simulation of $\mathbf{G}(t)$ with Caputo fractional operator with changing fractional values

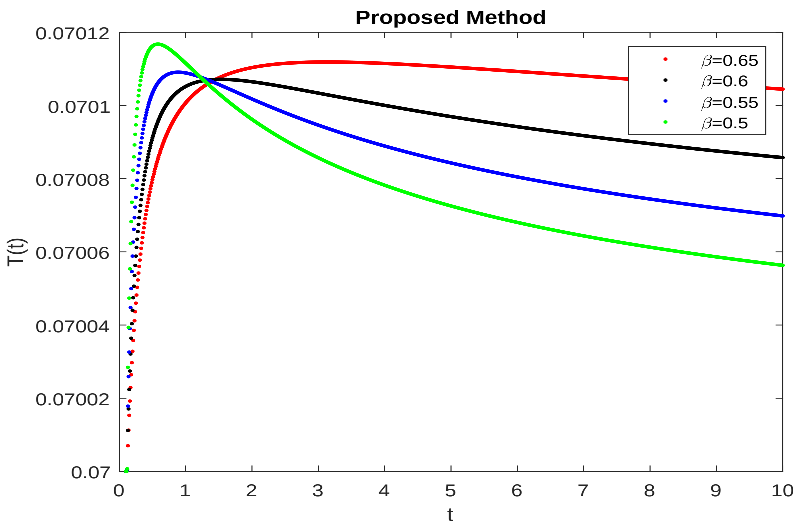

Figure 6: Simulation of $\mathbf{T}(t)$ with Caputo fractional operator with changing fractional values

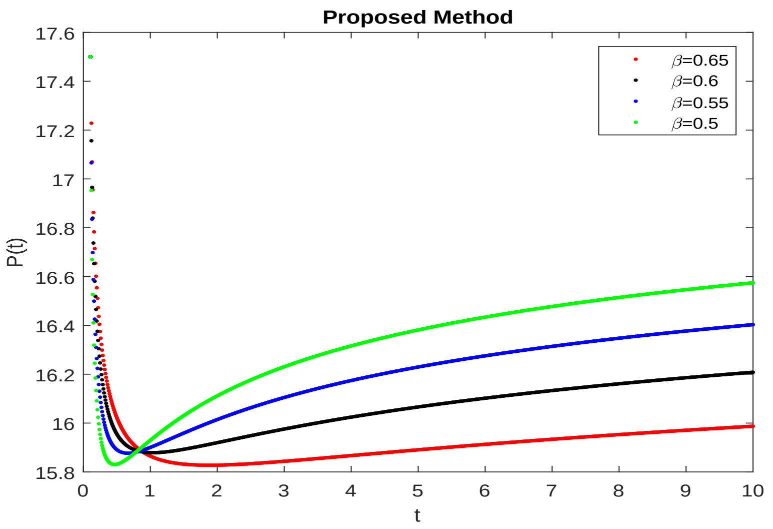

Figure 7: Simulation of $\mathbf{P}(t)$ with Caputo fractional operator with changing fractional values

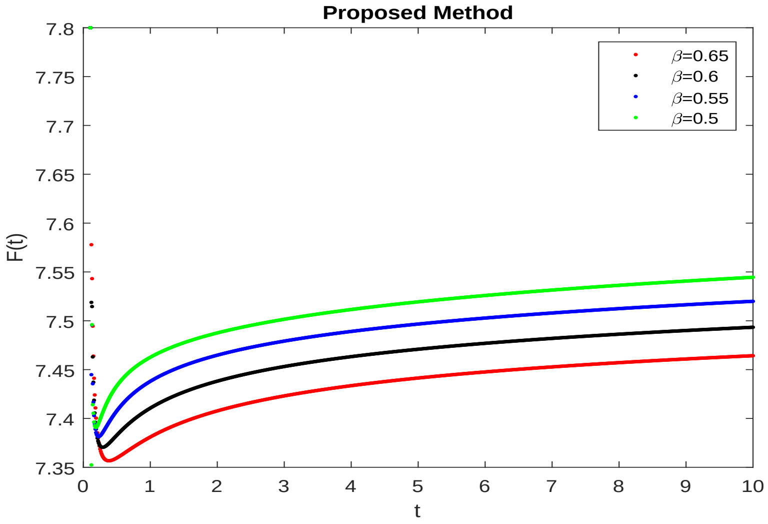

Figure 8: Simulation of $\mathbf{F}(t)$ with Caputo fractional operator with changing fractional values

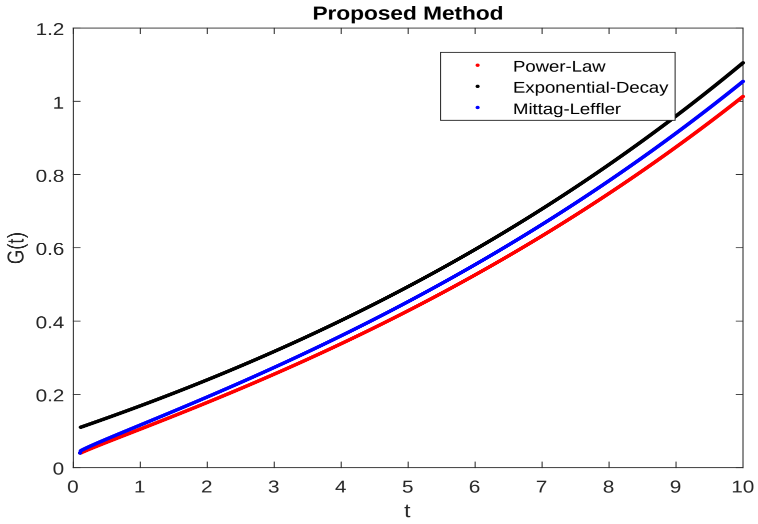

Figure 9: Simulation comparison of $\mathbf{G}(t)$ with different kernel under fractional operator $\beta = 0.9$

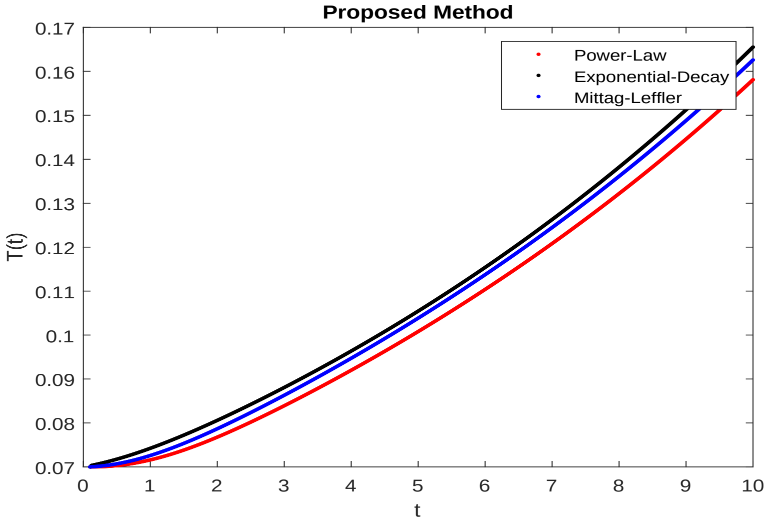

Figure 10: Simulation comparison of $\mathbf{T}(t)$ with different kernel under fractional operator $\beta = 0.9$

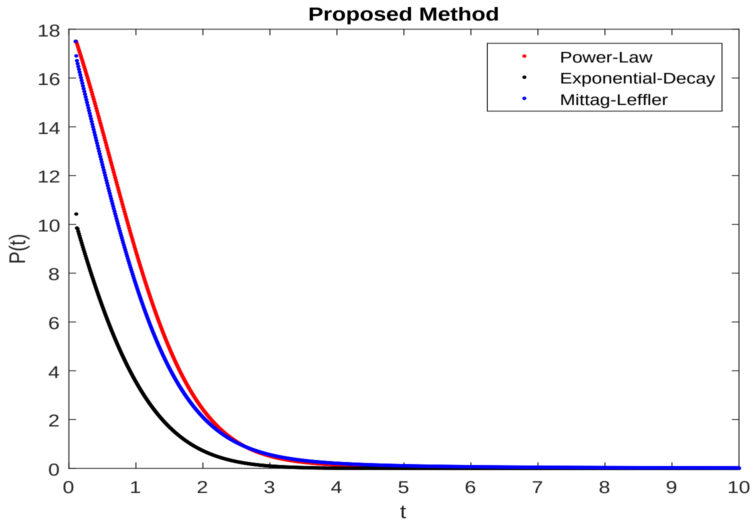

Figure 11: Simulation comparison of $\mathbf{P}(t)$ with different kernel under fractional operator $\beta = 0.9$

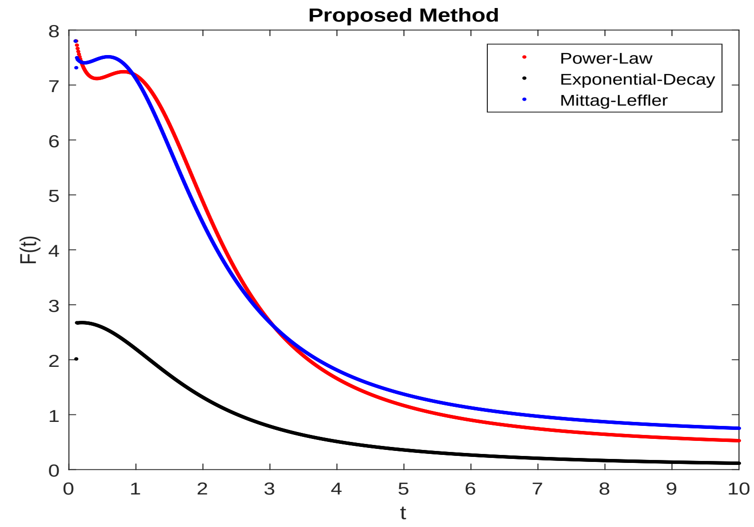

Figure 12: Simulation comparison of $\mathbf{F}(t)$ with different kernel under fractional operator $\beta = 0.9$

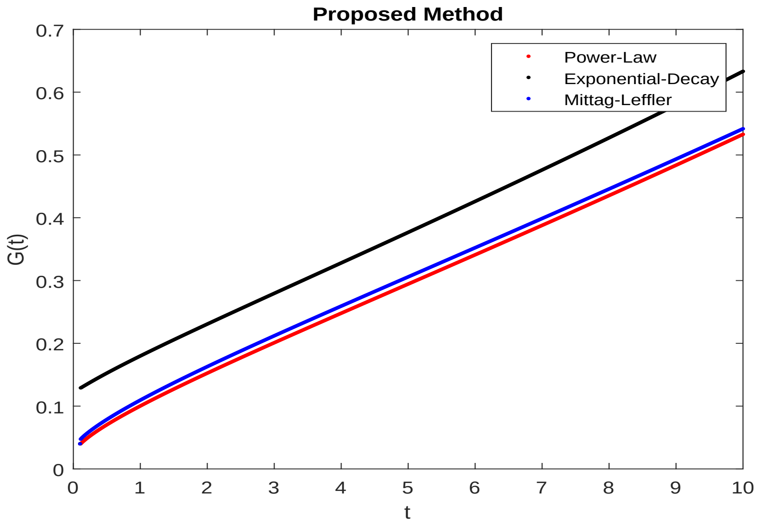

Figure 13: Simulation comparison of $\mathbf{G}(t)$ with different kernel under fractional operator $\beta = 0.8$

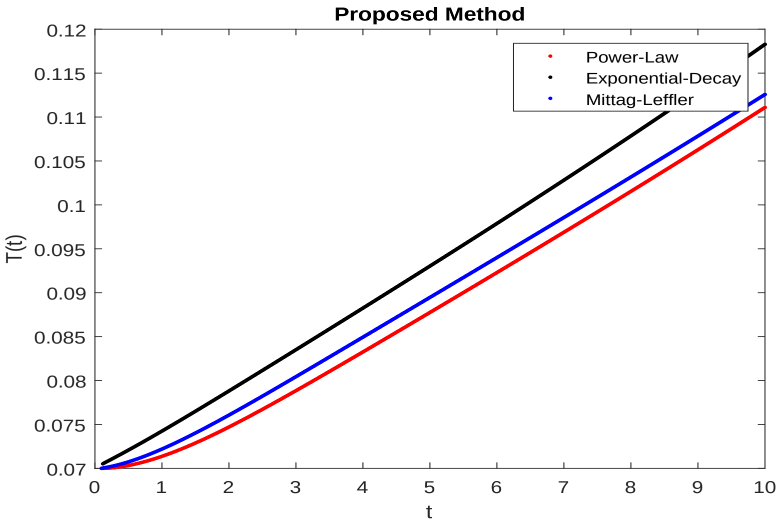

Figure 14: Simulation comparison of $\mathbf{T}(t)$ with different kernel under fractional operator $\beta = 0.8$

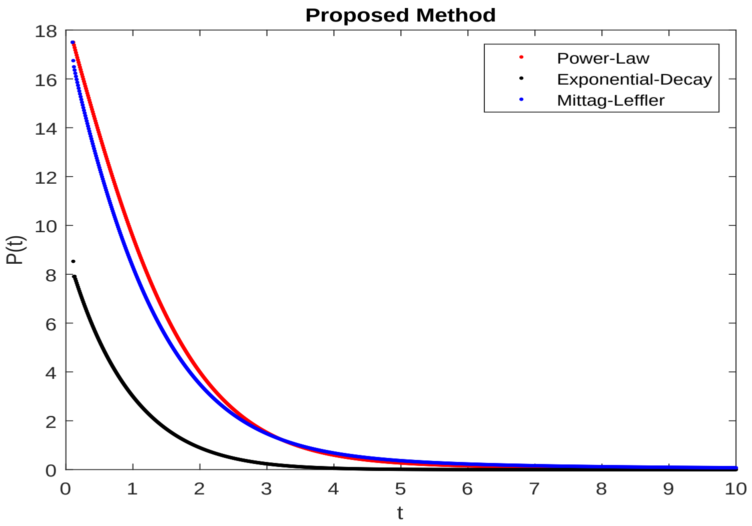

Figure 15: Simulation comparison of $\mathbf{P}(t)$ with different kernel under fractional operator $\beta = 0.8$

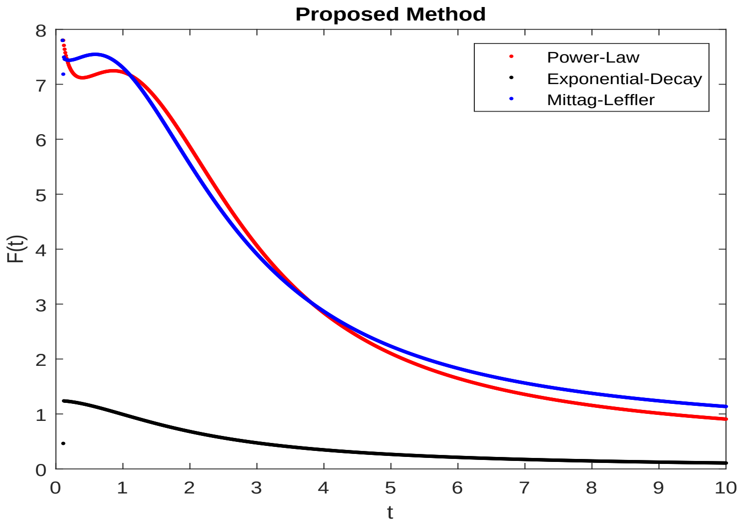

Figure 16: Simulation comparison of $\mathbf{F}(t)$ with different kernel under fractional operator $\beta = 0.8$

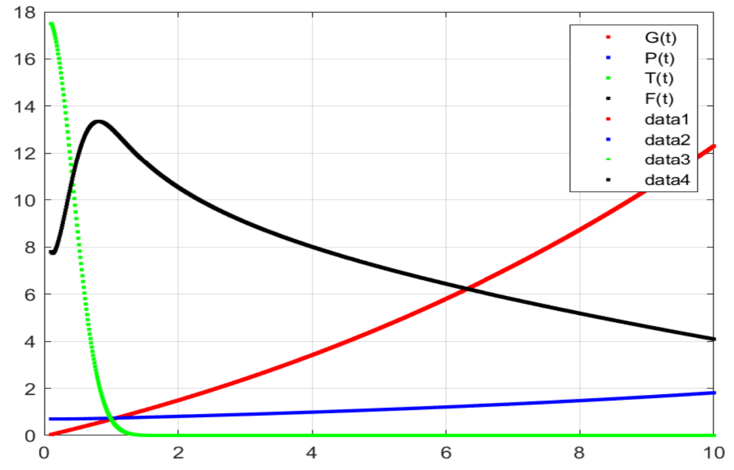

Figure 17: Simulation of all compartments in feasible region with chaotic form at fractional order 1

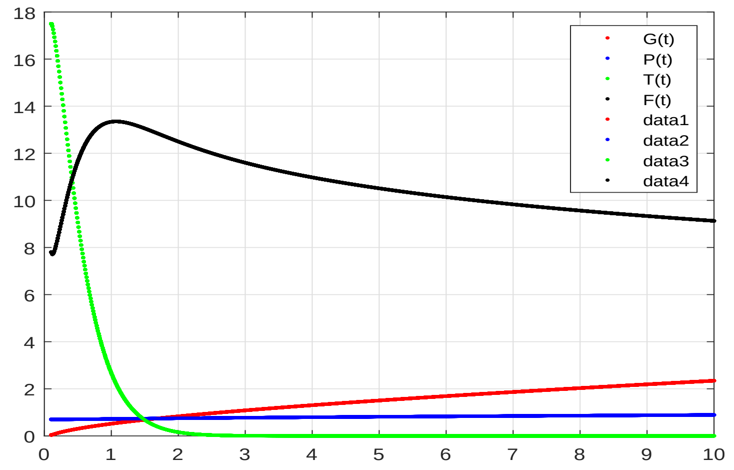

Figure 18: Simulation of all compartments in feasible region with chaotic form at fractional order 0.5

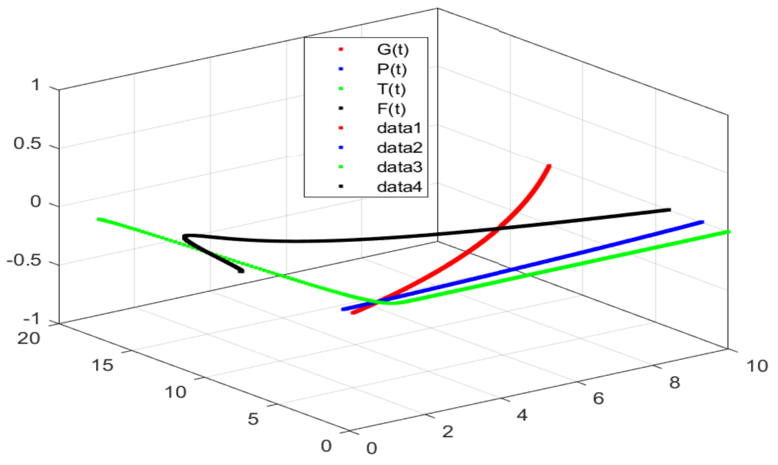

Figure 19: Simulation of all compartments in feasible region with chaotic form and bounded solution at different fractional value

6. Conclusion

In this study, we proposed a fractional order environment management model to research how rapidly accumulating ambient greenhouse gases (GGs) are affecting marine ecosystems and how this is contributing to global warming. We investigated the positively invariant region and showed that the model has positive, limited solutions. This can shed light on the resilience of these ecosystems and help in devising strategies for their conservation. To examine the model's existence and uniqueness, we also applied methods from several fixed-point theorems. Our results show the model to be generalized Ulam-Hyers-Rassias stable. The mathematical model was then resolved using a numerical method based on Newton's polynomial interpolation. Elaborating on the specific numerical techniques employed and the accuracy of the results would provide a better understanding of the model's practical utility. Results using different fractional values show significant variations. Non-integer order greatly impacts the flexibility and behavior of the solution curves, as is visible from the graphs. It indicates that small perturbations in the model's parameters do not lead to drastic changes in behavior, which is relevant for decision-making. This research can be expanded to include more generalized applicable fractional operators and improve control strategies for marine fisheries resources. To optimally utilize marine fisheries resources, effective measures attempt to increase plankton, minimize GGS concentration, and restrict worldwide warming. In the future, the study can be extended with some other parameters and fractional optimal control strategy.

Data Availability All data generated or analysed during this study are included in this published article
Conflict of interest The authors declare that they have no conflict of interest.

CRediT authorship contribution statement: : M.F.: Conceptualization, Methodology, Formal analysis,Writing-original draft, Writing review editing. A.S.: Conceptualization, Methodology, Formal analysis, Software, Visualization, Writing original draft, K.S.N: Methodology, Formal analysis, Software, Formatting, Writing review, editing. E.H.: Formal analysis, Supervision, Visualization, Writing-review , editing. A.A.: Formal analysis, Software, Visualization, Writing-review editing. A.M.H.: Methodology, Formal analysis, Visualization, Methodology,

References

[1] Sekerci, Yadigar, and Sergei Petrovskii. "Mathematical modelling of planktonoxygen dynamics under the climate change." Bulletin of mathematical biology 77 (2015): 2325-2353.

[2] Lv, Yunfei, Yongzhen Pei, Shujing Gao, and Changguo Li. "Harvesting of a phytoplanktonzooplankton model." Nonlinear Analysis: Real World Applications 11, no. 5 (2010): 3608-3619.

[3] Alvain, Séverine, Lucile Duforêt-Gaurier, and Hubert Loisel. "Observation of ocean colour beyond chlorophyll-a: From particulate organic carbon content and size distribution to phytoplankton functional groups." In: Handbook of Satellite Remote Sensing Image Interpretation: Applications for Marine Living Resources Conservation and Management (2011): 65-77.

[4] Winder, Monika, and Ulrich Sommer. "Phytoplankton response to a changing climate." Hydrobiologia 698 (2012): 5-16.

[5] Mandal, Sajib, Md Sirajul Islam, Md Haider Ali Biswas, and Sonia Akter. "A mathematical model applied to investigate the potential impact of global warming on marine ecosystems." Applied Mathematical Modelling 101 (2022): 19-37.

[6] Roxy, Mathew Koll, Aditi Modi, Raghu Murtugudde, Vinu Valsala, Swapna Panickal, S. Prasanna Kumar, M. Ravichandran, Marcello Vichi, and Marina Lévy. "A reduction in marine primary productivity driven by rapid warming over the tropical Indian Ocean." Geophysical Research Letters 43, no. 2 (2016): 826-833.

[7] Asch, Rebecca G., William WL Cheung, and Gabriel Reygondeau. "Future marine ecosystem drivers, biodiversity, and fisheries maximum catch potential in Pacific Island countries and territories under climate change." Marine Policy 88 (2018): 285-294.

[8] Celik, Senol. "The effects of climate change on human behaviors." Environment, climate, plant and vegetation growth (2020): 577-589.

[9] Waqih, Muhammad Abaid Ullah, Niaz Ahmed Bhutto, Niaz Hussain Ghumro, Suresh Kumar, and Muhammad Abdul Salam. "Rising environmental degradation and impact of foreign direct investment: an empirical evidence from SAARC region." Journal of environmental management 243 (2019): 472-480.

[10] Yu, Tong, and Yinguang Chen. "Effects of elevated carbon dioxide on environmental microbes and its mechanisms: A review." Science of The Total Environment 655 (2019): 865-879.

[11] Lüthi, Dieter, Martine Le Floch, Bernhard Bereiter, Thomas Blunier, Jean-Marc Barnola, Urs Siegenthaler, Dominique Raynaud et al. "High-resolution carbon dioxide concentration record 650,000800,000 years before present." nature 453, no. 7193 (2008): 379-382.

[12] Aronson, Richard B., Sven Thatje, James B. McClintock, and Kevin A. Hughes. "Anthropogenic impacts on marine ecosystems in Antarctica." Annals of the New York Academy of Sciences 1223, no. 1 (2011): 82-107.

[13] Bezirtzoglou, Christos, Konstantinos Dekas, and Ekatherina Charvalos. "Climate changes, environment and infection: facts, scenarios and growing awareness from the public health community within Europe." Anaerobe 17, no. 6 (2011): 337-340.

[14] Asch, Rebecca G., William WL Cheung, and Gabriel Reygondeau. "Future marine ecosystem drivers, biodiversity, and fisheries maximum catch potential in Pacific Island countries and territories under climate change." Marine Policy 88 (2018): 285-294.

[15] Baltar, Federico, Barbara Bayer, Nina Bednarsek, Stacy Deppeler, Ruben Escribano, Carolina E. Gonzalez, Roberta L. Hansman et al. "Towards integrating evolution, metabolism, and climate change studies of marine ecosystems." Trends in ecology & evolution 34, no. 11 (2019): 1022-1033.

[16] Speers, Ann E., Elena Y. Besedin, James E. Palardy, and Chris Moore. "Impacts of climate change and ocean acidification on coral reef fisheries: an integrated ecological-economic model." Ecological economics 128 (2016): 33-43.

[17] Ahmad, Aqeel, Maryam Shahid, Muhammad Farman, and M. O. Ahmad. "Stability and Convergence Analysis of Smoking Impact in Society with Algorithm Aspects." International Journal of Analysis and Applications 17, no. 4 (2019): 503-516.

[18] Kim, Seung-Uk, and Kwang-Yul Kim. "Impact of climate change on the primary production and related biogeochemical cycles in the coastal and sea ice zone of the Southern Ocean." Science of the Total Environment 751 (2021): 141678.

[19] Mandal, Sajib, Md Sirajul Islam, and Md Haider Ali Biswas. "Modeling and analytical analysis of the effect of atmospheric temperature to the planktonic ecosystem in oceans." In Applications of Internet of Things: Proceedings of ICCCIOT 2020, pp. 131-140. Springer Singapore, 2021.

[20] Speers, Ann E., Elena Y. Besedin, James E. Palardy, and Chris Moore. "Impacts of climate change and ocean acidification on coral reef fisheries: an integrated ecological-economic model." Ecological economics 128 (2016): 33-43.

[21] Häder, Donat-P., and Paul W. Barnes. “Comparing the impacts of climate change on the responses and linkages between terrestrial and aquatic ecosystems.” Science of the Total Environment 682 (2019): 239-246.

[22] Shen, W. Y., Chu, Y. M., ur Rahman, M., Mahariq, I., & Zeb, A. (2021). Mathematical analysis of HBV and HCV co-infection model under nonsingular fractional order derivative. Results in Physics, 28, 104582.

[23] Farman, Muhammad, Rabia Sarwar, Sameh Askar, Hijaz Ahmad, Muhammad Sultan, and Muhammad Manan Akram. “Fractional order model to study the impact of planting genetically modified trees on the regulation of atmospheric carbon dioxide with analysis and modeling.” Results in Physics 48 (2023): 106409.

[24] Nisar, Kottakkaran Sooppy, Muhammad Farman, Evren Hincal, and Aamir Shehzad. “Modelling and analysis of bad impact of smoking in society with Constant Proportional-Caputo Fabrizio operator.” Chaos, Solitons & Fractals 172 (2023): 113549.

[25] Jin, F., Qian, Z. S., Chu, Y. M., & ur Rahman, M. (2022). On nonlinear evolution model for drinking behavior under Caputo-Fabrizio derivative. J. Appl. Anal. Comput, 12(2), 790-806.

[26] Chu, Y. M., Khan, M. F., Ullah, S., Shah, S. A. A., Farooq, M., & bin Mamat, M. (2023). Mathematical assessment of a fractional-order vectorhost disease model with the CaputoFabrizio derivative. Mathematical Methods in the Applied Sciences, 46(1), 232-247.

[27] Sekerci, Yadigar, and Ramazan Ozarslan. “Dynamic analysis of time fractional order oxygen in a plankton system.” The European Physical Journal Plus 135, no. 1 (2020): 88.

[28] Ozarslan, Ramazan, and Yadigar Sekerci. “Fractional order oxygen-plankton system under climate change.” Chaos: An Interdisciplinary Journal of Nonlinear Science 30, no. 3 (2020).

[29] Shi, Ruiqing, Jianing Ren, and Cuihong Wang. “Stability analysis and Hopf bifurcation of a fractional order mathematical model with time delay for nutrient-phytoplankton-zooplankton.” Mathematical Biosciences and Engineering 17, no. 4 (2020): 3836-3868.

[30] Agarwal, Praveen, Ashraf Adnan Thirthar, and Abdesslem Lamrani Alaoui. “The Exponential Decay Functions for Prey and Predator Species in a Fractional-Order Ecosystem Model with Considering Global Warming Phenomena.” Authorea Preprints (2022).

[31] Bonyah, Ebenezer. “Fractional Optimal Control Model for Nutrients, Phytoplankton, and Zooplankton.” In Applications of Fractional Calculus to Modeling in Dynamics and Chaos, pp. 429-452. Chapman and Hall/CRC, 2022.

[32] Kumar, Pushpendra, Vedat Suat Erturk, Ramashis Banerjee, Mehmet Yavuz, and V. Govindaraj. “Fractional modeling of plankton-oxygen dynamics under climate change by the application of a recent numerical algorithm.” Physica Scripta 96, no. 12 (2021): 124044.

[33] Kumar, Pushpendra, V. Govindaraj, Vedat Suat Erturk, and Mohamed S. Mohamed. “Effects of greenhouse gases and hypoxia on the population of aquatic species: a fractional mathematical model.” Advances in Continuous and Discrete Models 2022, no. 1 (2022): 1-19.

[34] Caputo, Michele. “Linear models of dissipation whose Q is almost frequency independent-II.” Geophysical Journal International 13, no. 5 (1967): 529-539.

[35] Farman, Muhammad, Cicik Alfiniyah, and Aamir Shehzad. “Modelling and analysis tuberculosis (TB) model with hybrid fractional operator.” Alexandria Engineering Journal 72 (2023): 463-478.

[36] Vargas-De-León, Cruz. "Volterra-type Lyapunov functions for fractional-order epidemic systems." Communications in Nonlinear Science and Numerical Simulation 24, no. 1-3 (2015): 75-85.

[37] Atangana, Abdon. "Mathematical model of survival of fractional calculus, critics and their impact: How singular is our world?." Advances in Difference Equations 2021, no. 1 (2021): 1-59.

[38] Odionyenma, Udoka Benedict, Nometa Ikenna, and Bolarinwa Bolaji. "Analysis of a model to control the co-dynamics of Chlamydia and Gonorrhea using Caputo fractional derivative." Mathematical Modelling and Numerical Simulation with Applications 3, no. 2 (2023): 111-140.

[39] Liu, K., Feckan, M., & Wang, J. (2020). HyersUlam stability and existence of solutions to the generalized LiouvilleCaputo fractional differential equations. Symmetry, 12(6), 955.
