# Supplementary figures and images for: Generalized Ulam-Hyers-Rassias stability and novel sustainable techniques for dynamical analysis of global warming impact on ecosystem
Source: Sci Rep. 2023 Dec 17;13:22441. doi: 10.1038/s41598-023-49806-7 (PMC10725897; doi:10.1038/s41598-023-49806-7)

# Proposed Method

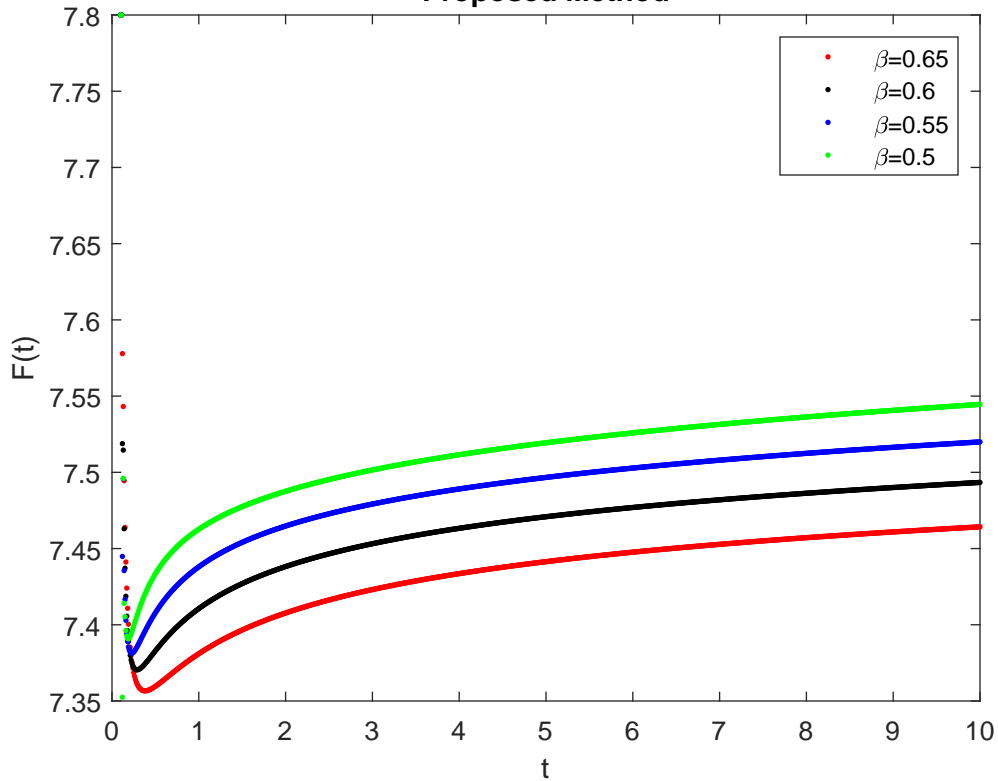

Supplement: Supplementary file 1 — Supplementary Information. [file 41598_2023_49806_MOESM1_ESM.zip › Revised Manuscript (1)/Revised Manuscript/5F-eps-converted-to.pdf]

# Proposed Method

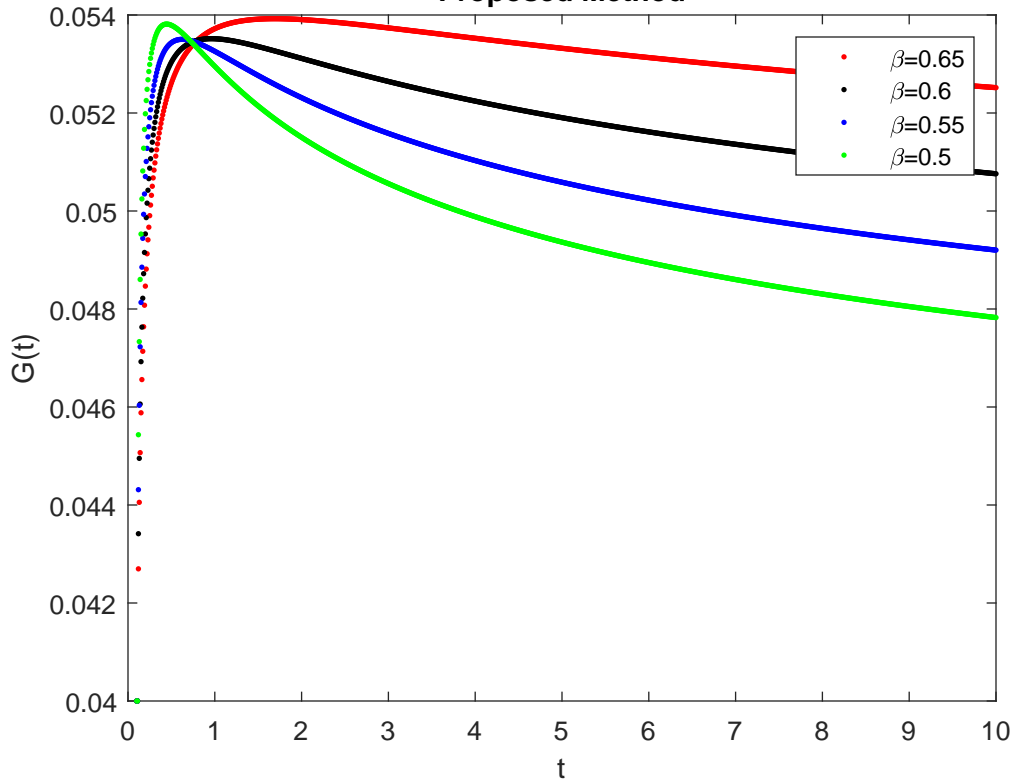

Supplement: Supplementary file 1 — Supplementary Information. [file 41598_2023_49806_MOESM1_ESM.zip › Revised Manuscript (1)/Revised Manuscript/5G-eps-converted-to.pdf]

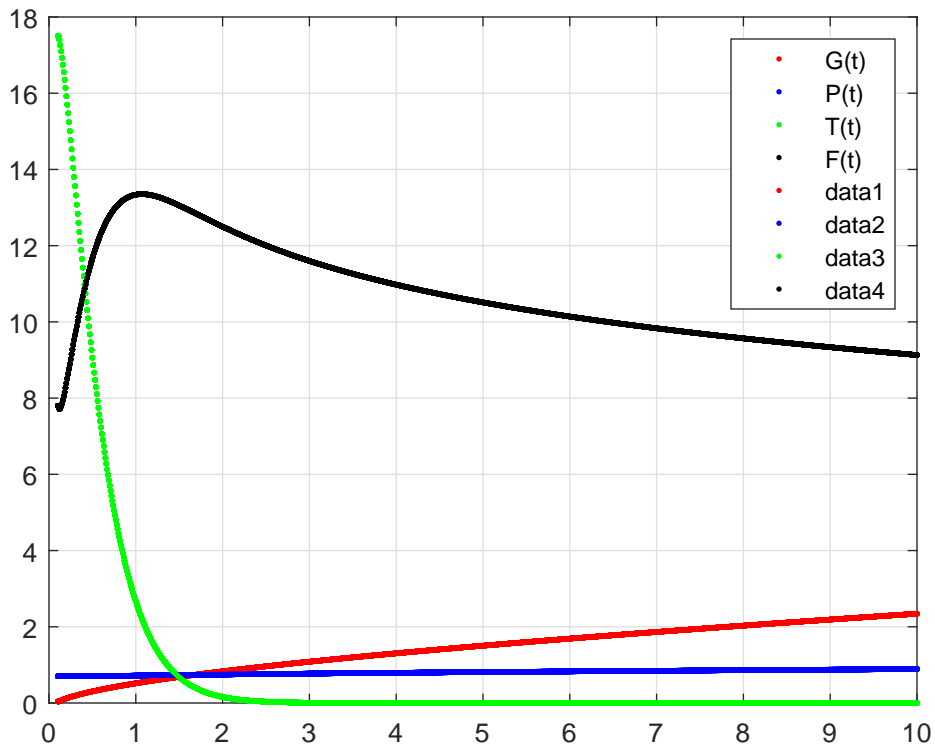

Supplement: Supplementary file 1 — Supplementary Information. [file 41598_2023_49806_MOESM1_ESM.zip › Revised Manuscript (1)/Revised Manuscript/5gptf-eps-converted-to.pdf]

# Proposed Method

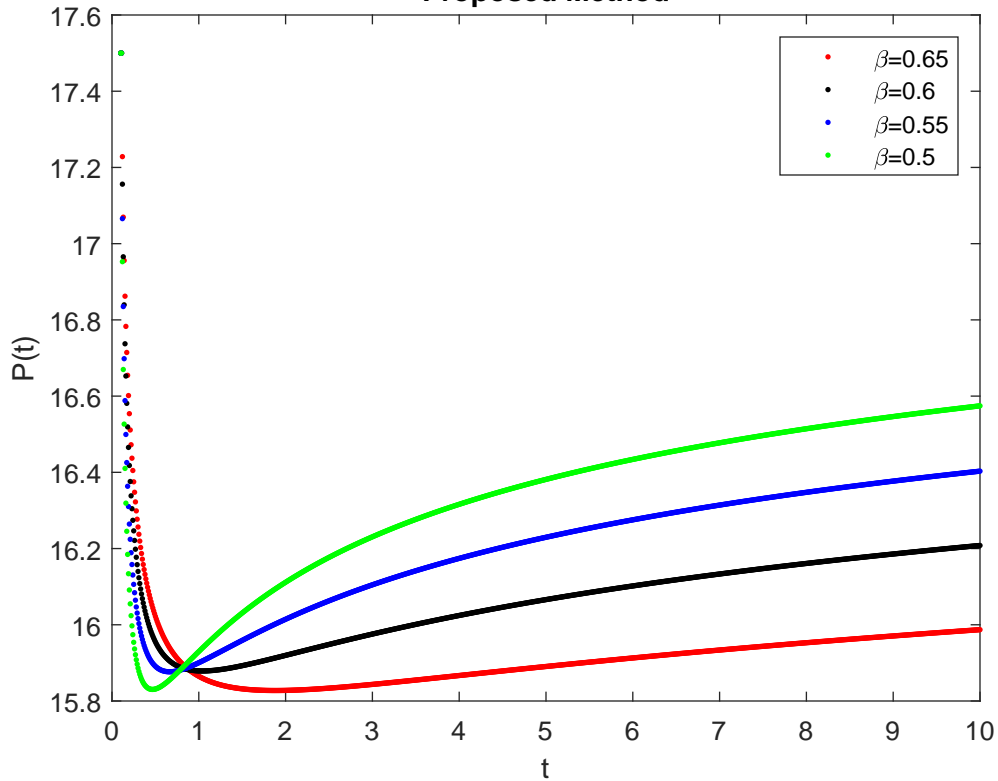

Supplement: Supplementary file 1 — Supplementary Information. [file 41598_2023_49806_MOESM1_ESM.zip › Revised Manuscript (1)/Revised Manuscript/5P-eps-converted-to.pdf]

# Proposed Method

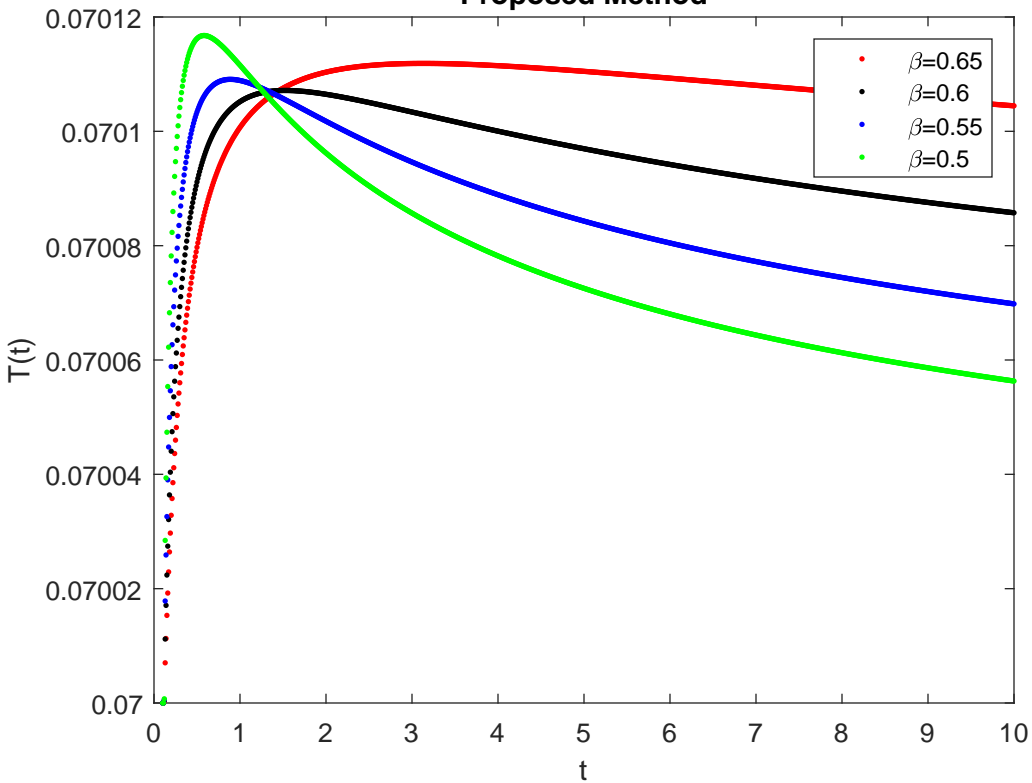

Supplement: Supplementary file 1 — Supplementary Information. [file 41598_2023_49806_MOESM1_ESM.zip › Revised Manuscript (1)/Revised Manuscript/5T-eps-converted-to.pdf]

## Proposed Method

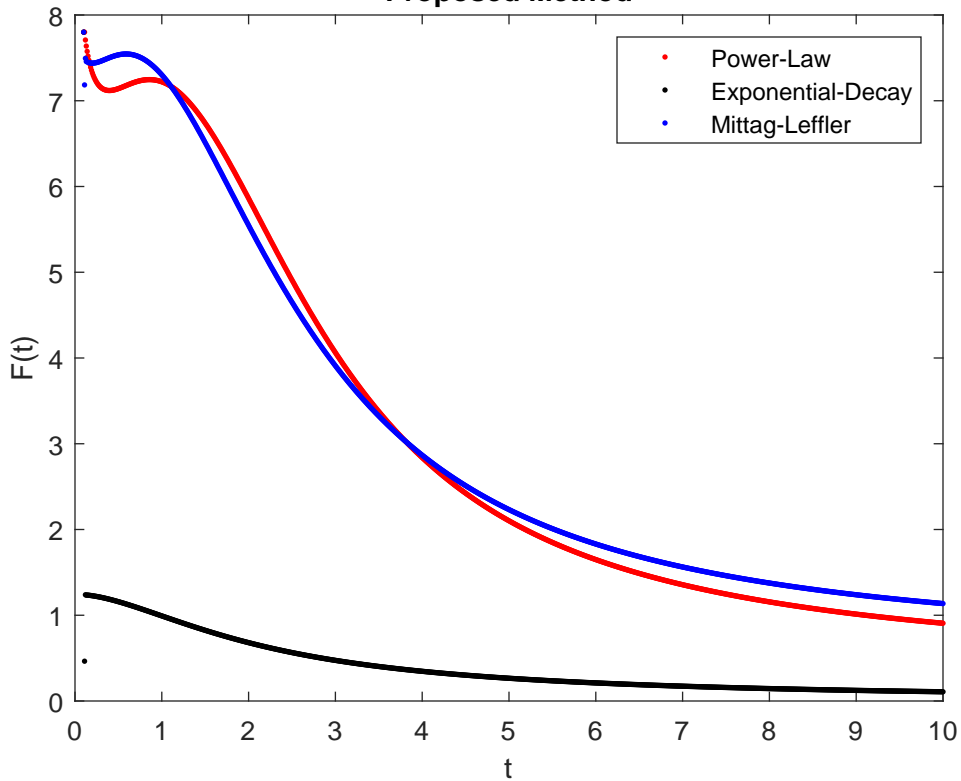

Supplement: Supplementary file 1 — Supplementary Information. [file 41598_2023_49806_MOESM1_ESM.zip › Revised Manuscript (1)/Revised Manuscript/8F1-eps-converted-to.pdf]

## Proposed Method

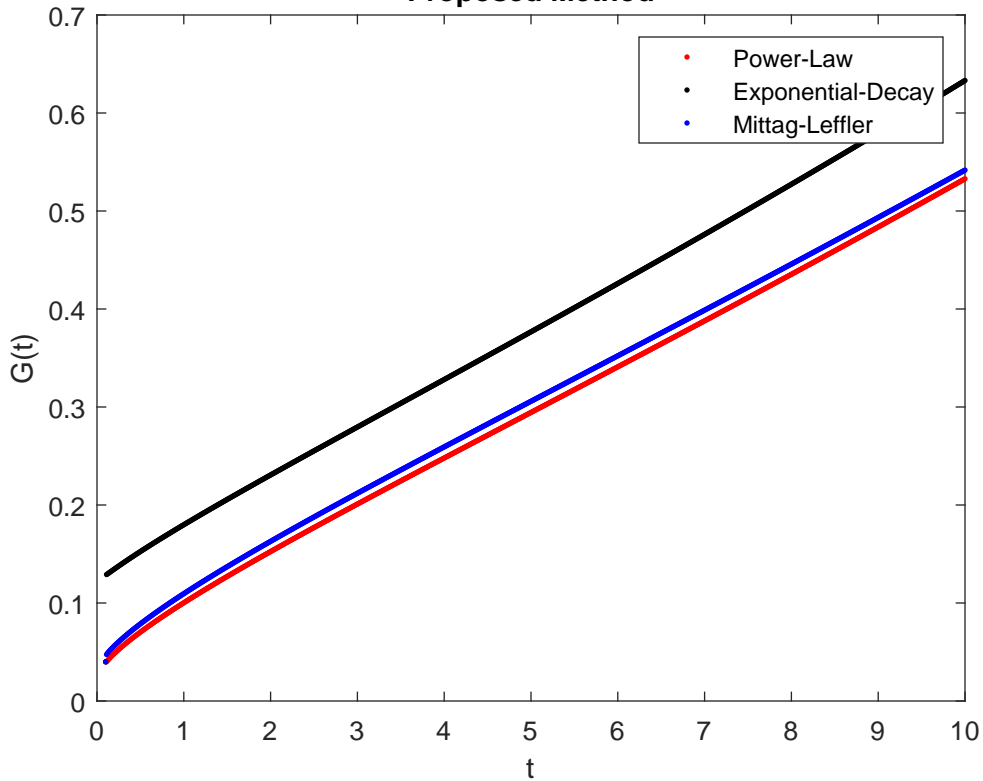

Supplement: Supplementary file 1 — Supplementary Information. [file 41598_2023_49806_MOESM1_ESM.zip › Revised Manuscript (1)/Revised Manuscript/8G1-eps-converted-to.pdf]

## Proposed Method

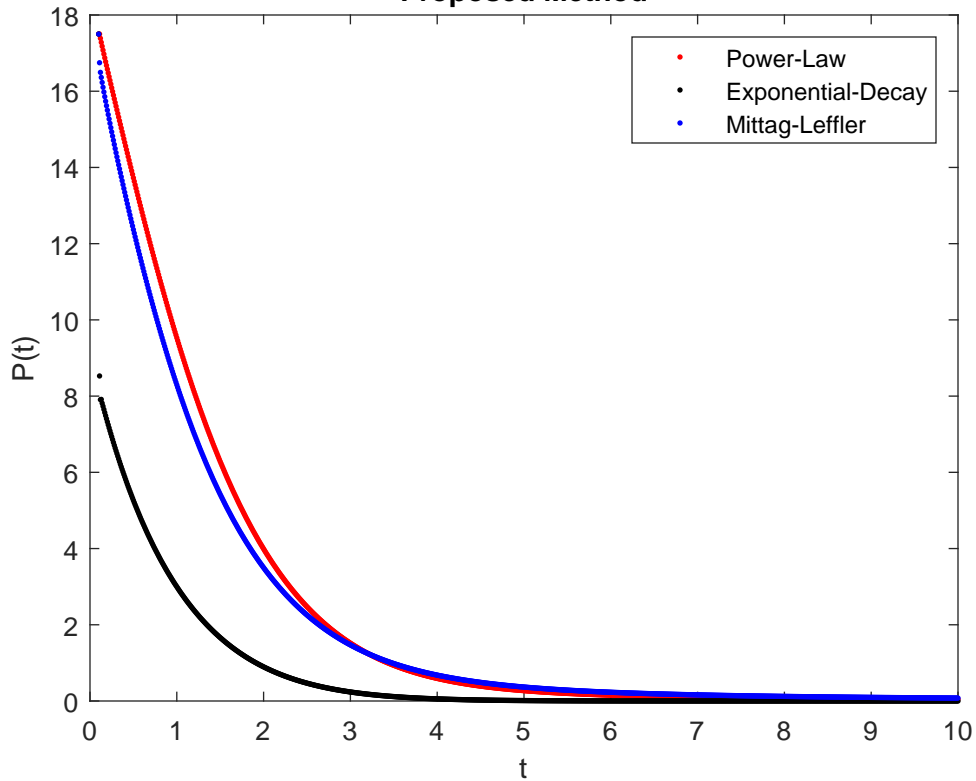

Supplement: Supplementary file 1 — Supplementary Information. [file 41598_2023_49806_MOESM1_ESM.zip › Revised Manuscript (1)/Revised Manuscript/8P1-eps-converted-to.pdf]

## Proposed Method

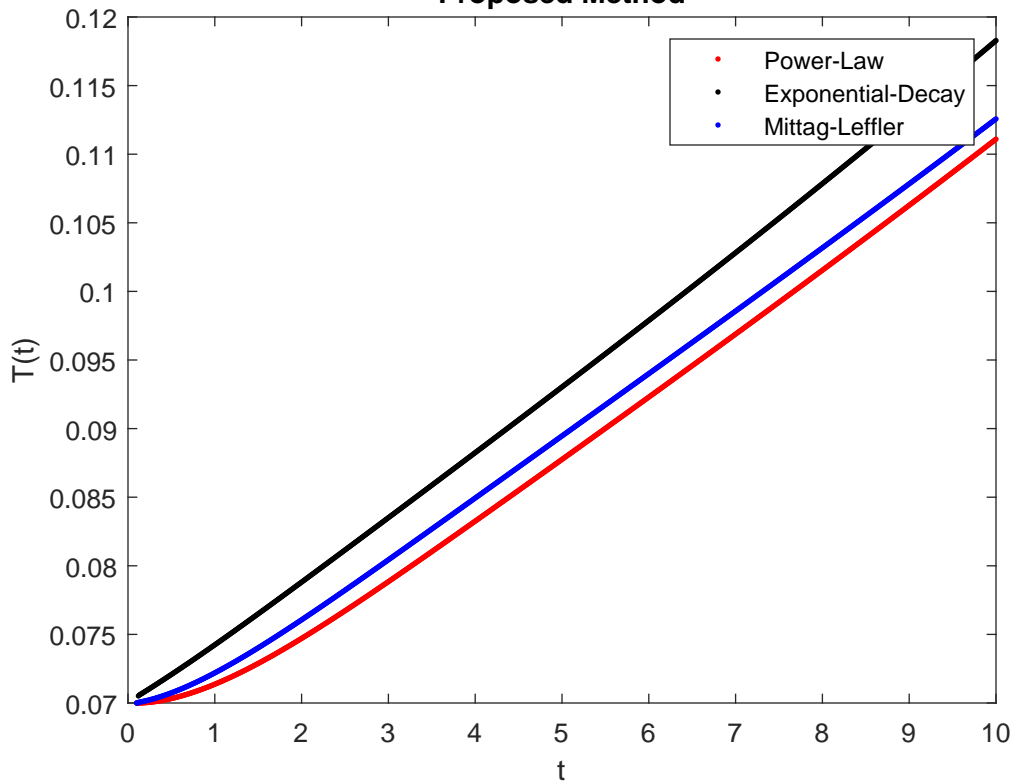

Supplement: Supplementary file 1 — Supplementary Information. [file 41598_2023_49806_MOESM1_ESM.zip › Revised Manuscript (1)/Revised Manuscript/8T1-eps-converted-to.pdf]

## Proposed Method

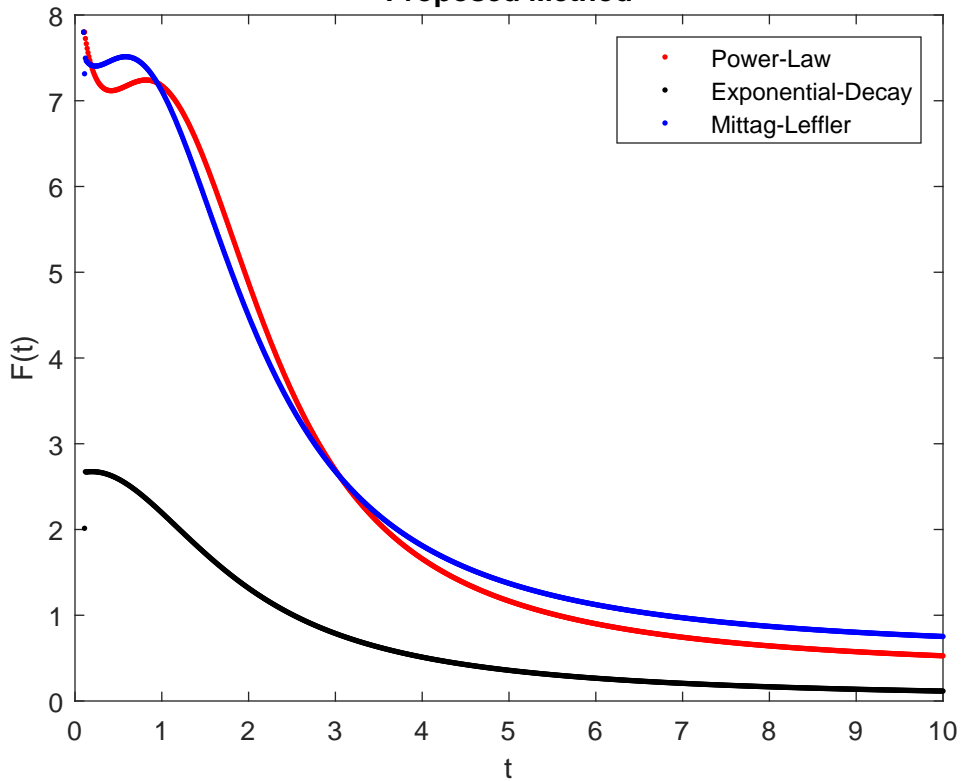

Supplement: Supplementary file 1 — Supplementary Information. [file 41598_2023_49806_MOESM1_ESM.zip › Revised Manuscript (1)/Revised Manuscript/F1-eps-converted-to.pdf]

# Proposed Method

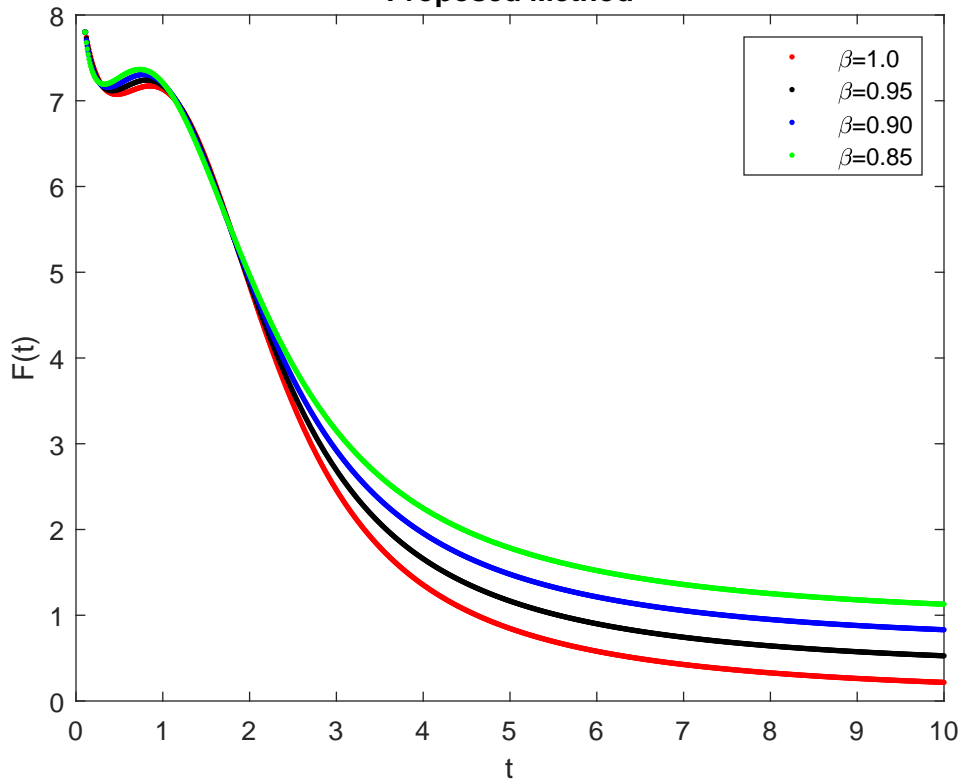

Supplement: Supplementary file 1 — Supplementary Information. [file 41598_2023_49806_MOESM1_ESM.zip › Revised Manuscript (1)/Revised Manuscript/F-eps-converted-to.pdf]

## Proposed Method

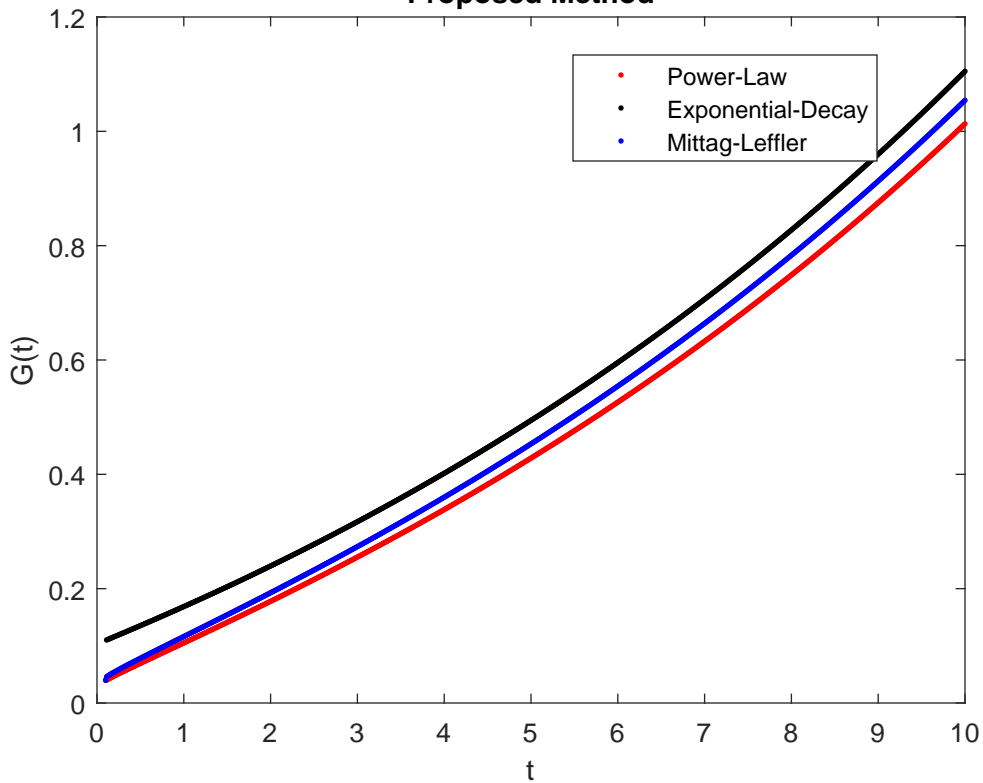

Supplement: Supplementary file 1 — Supplementary Information. [file 41598_2023_49806_MOESM1_ESM.zip › Revised Manuscript (1)/Revised Manuscript/G1-eps-converted-to.pdf]

**Proposed Method**

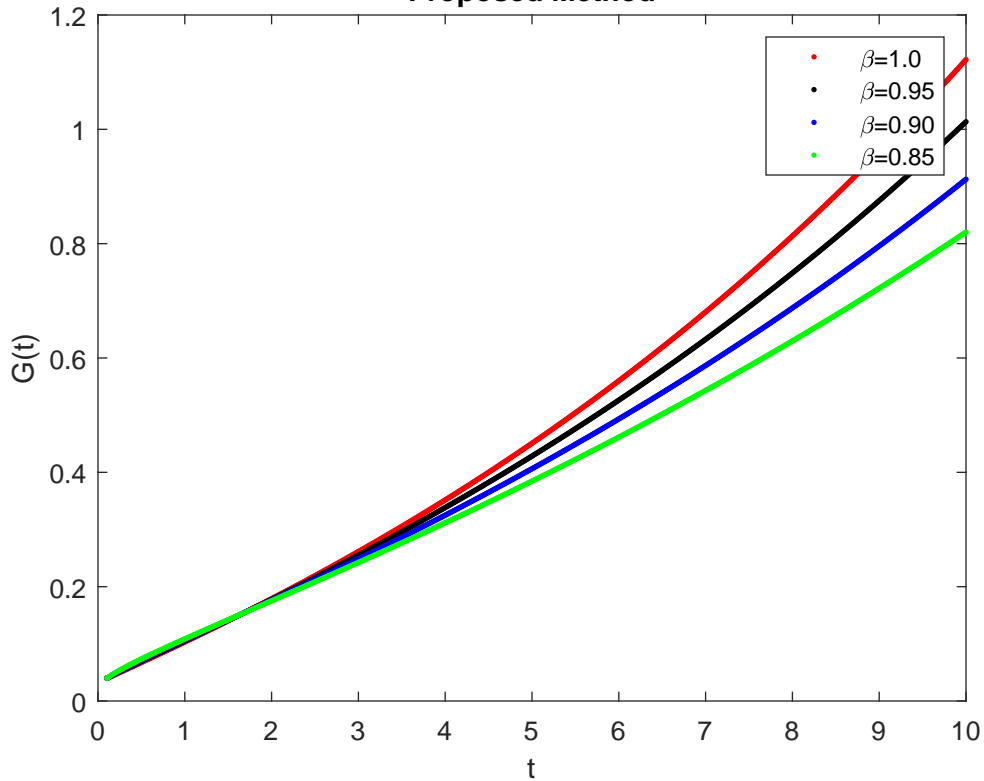

Supplement: Supplementary file 1 — Supplementary Information. [file 41598_2023_49806_MOESM1_ESM.zip › Revised Manuscript (1)/Revised Manuscript/G-eps-converted-to.pdf]

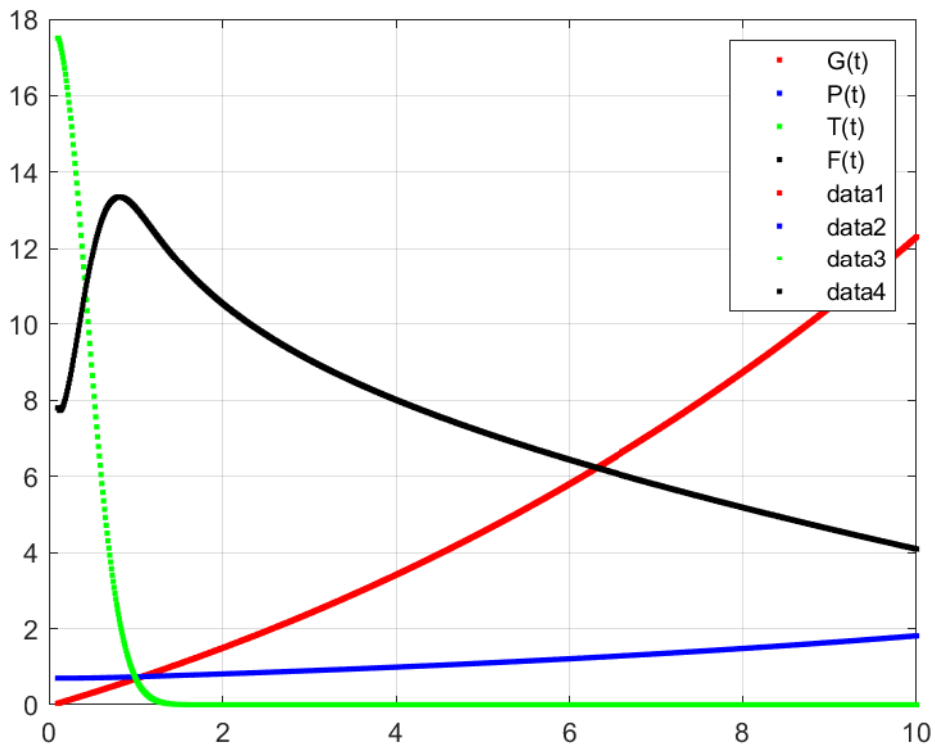

Supplement: Supplementary file 1 — Supplementary Information. [file 41598_2023_49806_MOESM1_ESM.zip › Revised Manuscript (1)/Revised Manuscript/gpft1-eps-converted-to.pdf]

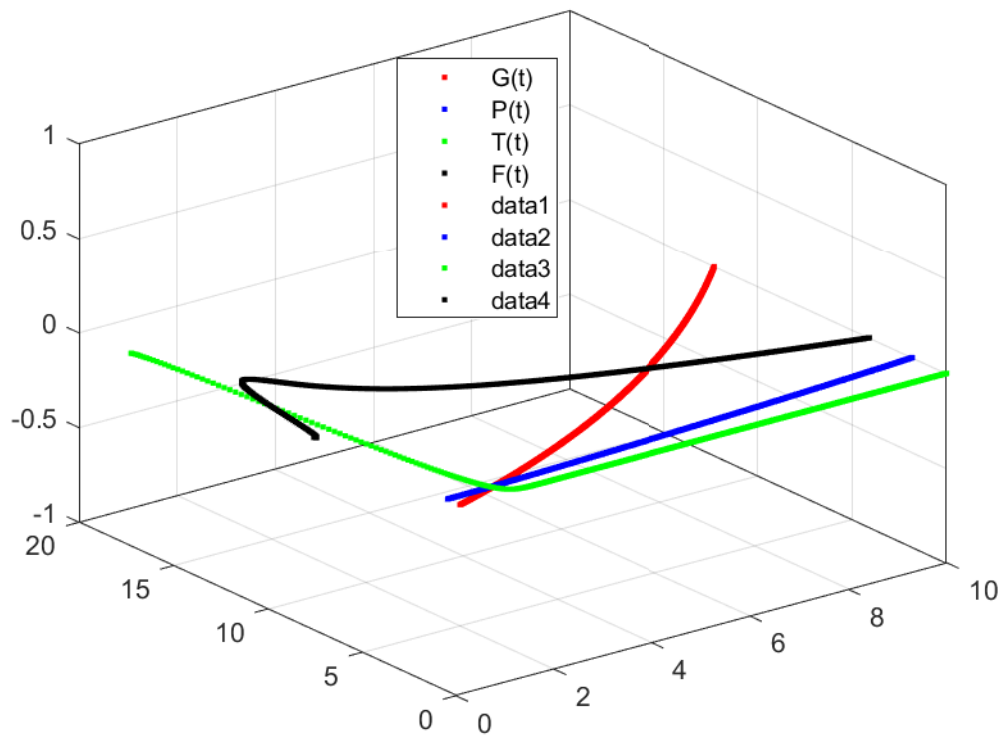

Supplement: Supplementary file 1 — Supplementary Information. [file 41598_2023_49806_MOESM1_ESM.zip › Revised Manuscript (1)/Revised Manuscript/gpft-eps-converted-to.pdf]

## Proposed Method

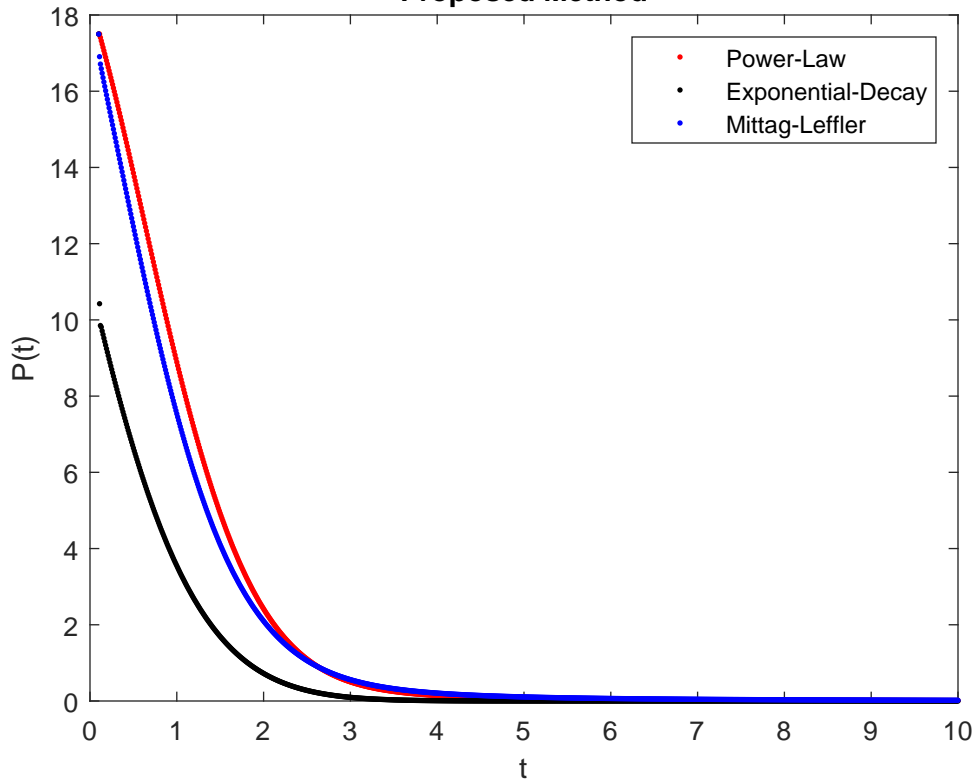

Supplement: Supplementary file 1 — Supplementary Information. [file 41598_2023_49806_MOESM1_ESM.zip › Revised Manuscript (1)/Revised Manuscript/P1-eps-converted-to.pdf]

# Proposed Method

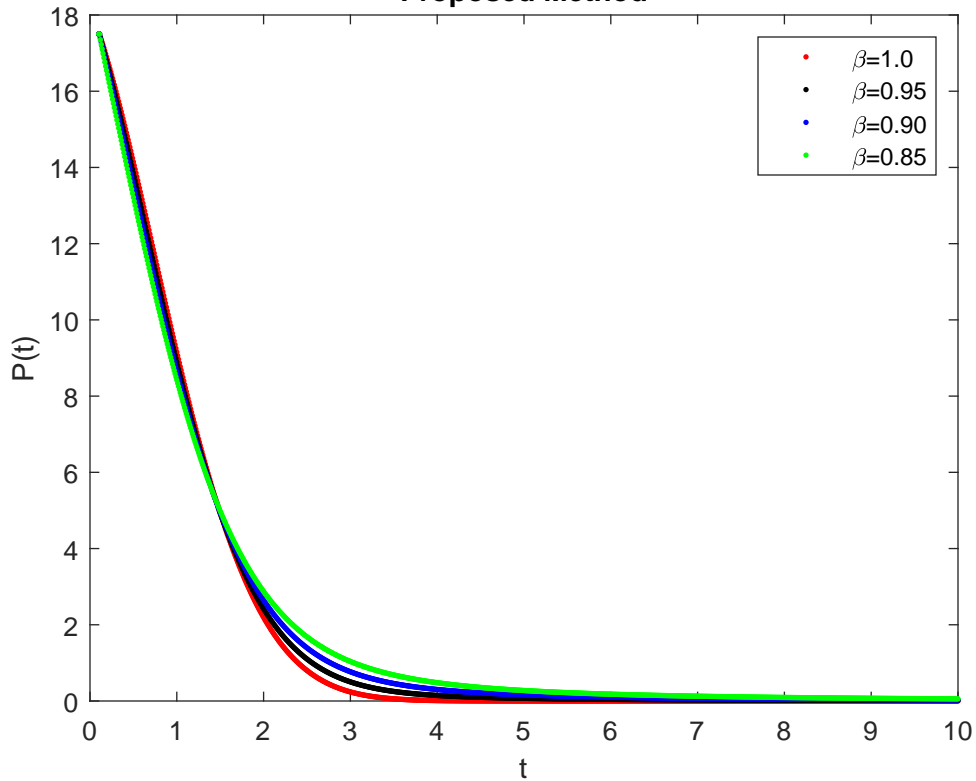

Supplement: Supplementary file 1 — Supplementary Information. [file 41598_2023_49806_MOESM1_ESM.zip › Revised Manuscript (1)/Revised Manuscript/P-eps-converted-to.pdf]

## Proposed Method

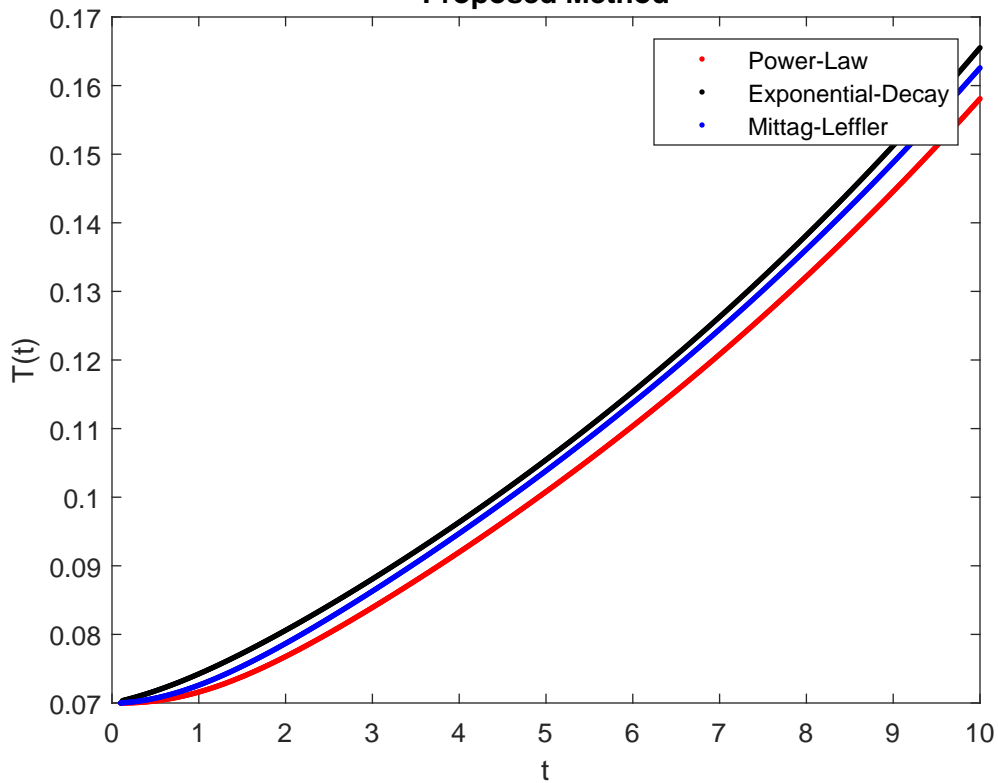

Supplement: Supplementary file 1 — Supplementary Information. [file 41598_2023_49806_MOESM1_ESM.zip › Revised Manuscript (1)/Revised Manuscript/T1-eps-converted-to.pdf]

# Proposed Method

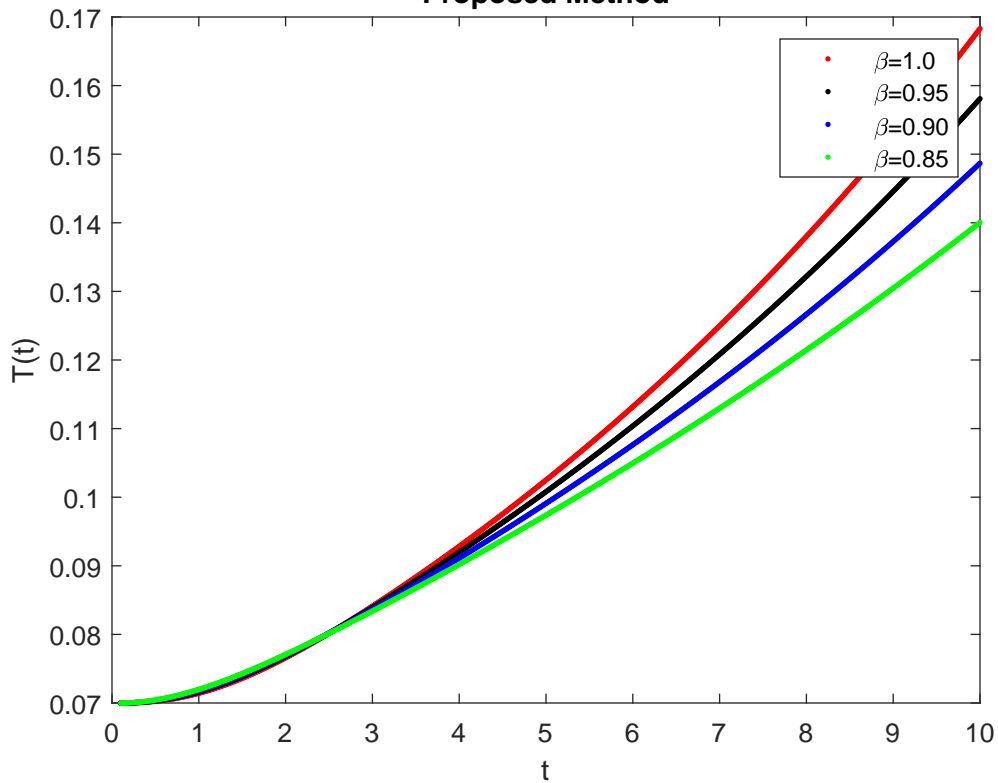

Supplement: Supplementary file 1 — Supplementary Information. [file 41598_2023_49806_MOESM1_ESM.zip › Revised Manuscript (1)/Revised Manuscript/T-eps-converted-to.pdf]
